# Digital screener of socio-motor agency balancing motor autonomy and motor control

**DOI:** 10.3389/fnhum.2024.1442799

**Published:** 2024-10-01

**Authors:** Theodoros Bermperidis, Richa Rai, Elizabeth B. Torres

**Affiliations:** ^1^Sensory Motor Integration Lab, Psychology Department, Rutgers University, Piscataway, NJ, United States; ^2^Rutgers University Center for Cognitive Science, Piscataway, NJ, United States; ^3^Computer Science Department, Rutgers University Center for Biomedicine Imaging and Modelling, Piscataway, NJ, United States

**Keywords:** autism, socio-motor agency, autonomy, control, entropy, stochastic analyses, signal-to-noise ratio, wearables

## Abstract

Dyadic social interactions evoke complex dynamics between two agents that, while exchanging unequal levels of body autonomy and motor control, may find a fine balance to synergize, take turns, and gradually build social rapport. To study the evolution of such complex interactions, we currently rely exclusively on subjective pencil and paper means. Here, we complement this approach with objective biometrics of socio-motor behaviors conducive to socio-motor agency. Using a common clinical test as the backdrop of our study to probe social interactions between a child and a clinician, we demonstrate new ways to streamline the detection of social readiness potential in both typically developing and autistic children by uncovering a handful of tasks that enable quantification of levels of motor autonomy and levels of motor control. Using these biometrics of autonomy and control, we further highlight differences between males and females and uncover a new data type amenable to generalizing our results to any social setting. The new methods convert continuous dyadic bodily biorhythmic activity into spike trains and demonstrate that in the context of dyadic behavioral analyses, they are well characterized by a continuous Gamma process that can classify individual levels of our thus defined socio-motor agency during a dyadic exchange. Finally, we apply signal detection processing tools in a machine learning approach to show the validity of the streamlined version of the digitized ADOS test. We offer a new framework that combines stochastic analyses, non-linear dynamics, and information theory to streamline and facilitate scaling the screening and tracking of social interactions with applications to autism.

## 1 Introduction

All research involving autism is (arguably) fundamentally tied to the Autism Diagnosis Observation Schedule [ADOS, currently in version 2 (Lord et al., 2000; Gotham et al., 2007; Torres et al., 2013a,b)]. Research spanning disparate fields, from genomics to complex social interactions, relies on this test as the gold standard to classify humans across the lifespan as autistic or on the autism spectrum. Although clinically validated, the ADOS-based diagnosis misses females (D'Mello et al., 2022; Lundstrom et al., 2019; Loomes et al., 2017). Moreover, there are not enough raters to absorb the large number of toddlers, children, and adults who, according to various screening tools, are suspected of being autistic today. The test is long and taxing on both the child and the clinician administering it because it has an average of 27 tasks aimed at engaging the child through social presses and expecting overtures from the child.

The ADOS is a dynamic and flexible test in the sense that the clinician can choose the tasks according to observing the flow of the child’s performance. It also adapts the test on demand, choosing the module that best agrees with the child’s communication level. However, the interaction occurs while the clinician also scores the child’s performance. Though valid to probe social competence, many of the tasks artificially rob the child of a chance to be naturally social, as the interaction is also taxing on the clinician and, at times, awkward and seemingly forced. In this sense, several of the tasks might be biased, interfering with the child’s socio-motor agency and robbing the clinician of the spontaneity characteristic of a natural social exchange. In this sense, we need objective ways to help automate the scoring process and to quantify this potential bias that such a taxing effect may produce on both social agents. New digital means could help us achieve such goals of automation and objectivity, but to be valid, they would need to preserve the clinical criteria, which have been empirically validated across decades. In this sense, clinically informed digital biomarkers that unveil physiological underpinnings of social and communication differences across neurodevelopment could be of use to both clinicians and researchers in the field of autism.

Prior work analyzing thousands of ADOS score records found non-obvious issues with the statistical foundations used to validate this test. While there are theoretical requirements of normality and homogeneous variance in the signal detection theory used to validate the ADOS (Somoza and Mossman, 1991; Hollander and Pena, 2004), as these assumptions are required for independence between bias and sensitivity (Torres et al., 2020b), and to decrease false positives, the empirical data across thousands of records violate these assumptions (Torres et al., 2020b). New methods have then been proposed to help reduce the number of tasks (Bokadia et al., 2020), while also utilizing motor signatures to identify females (Torres et al., 2013b, 2016a, 2017; Bokadia et al., 2020). Several studies related to the sense of agency have addressed interactions in the context of human–computer interfaces (Weiss et al., 2014; Cornelio et al., 2022). However, there are no means to define naturalistic socio-motor agency during physical dyadic interactions and to identify agency in tasks that enhance autonomy in autism (Torres et al., 2013c). Furthermore, no means to implement these tasks using artificial intelligence (AI) and machine learning (ML) methods have been proposed. Such approaches would help us speed up, automate, and scale the assessment process, particularly doing so with respect to currently underdiagnosed females (D'Mello et al., 2022). Moreover, the automated approaches could help us bring the diagnosis to rural and underserved areas, thus helping us diversify our research pool and enrich our therapies.

We reasoned in the present study that the digital ADOS (Bokadia et al., 2020), i.e., the ADOS that is digitally recorded while the child and clinician interact, could leverage the validity of this test as the gold standard for clinical and research use while providing a streamlined version of it that could help us (1) identify objective biometrics of socio-motor agency and (2) automate the process of identifying socially compliant tasks [as defined by the ADOS test (Lord et al., 2000; Gotham et al., 2007; Torres et al., 2013a,b)] using methods from artificial intelligence (AI) and machine learning (ML).

**Figure 1:**
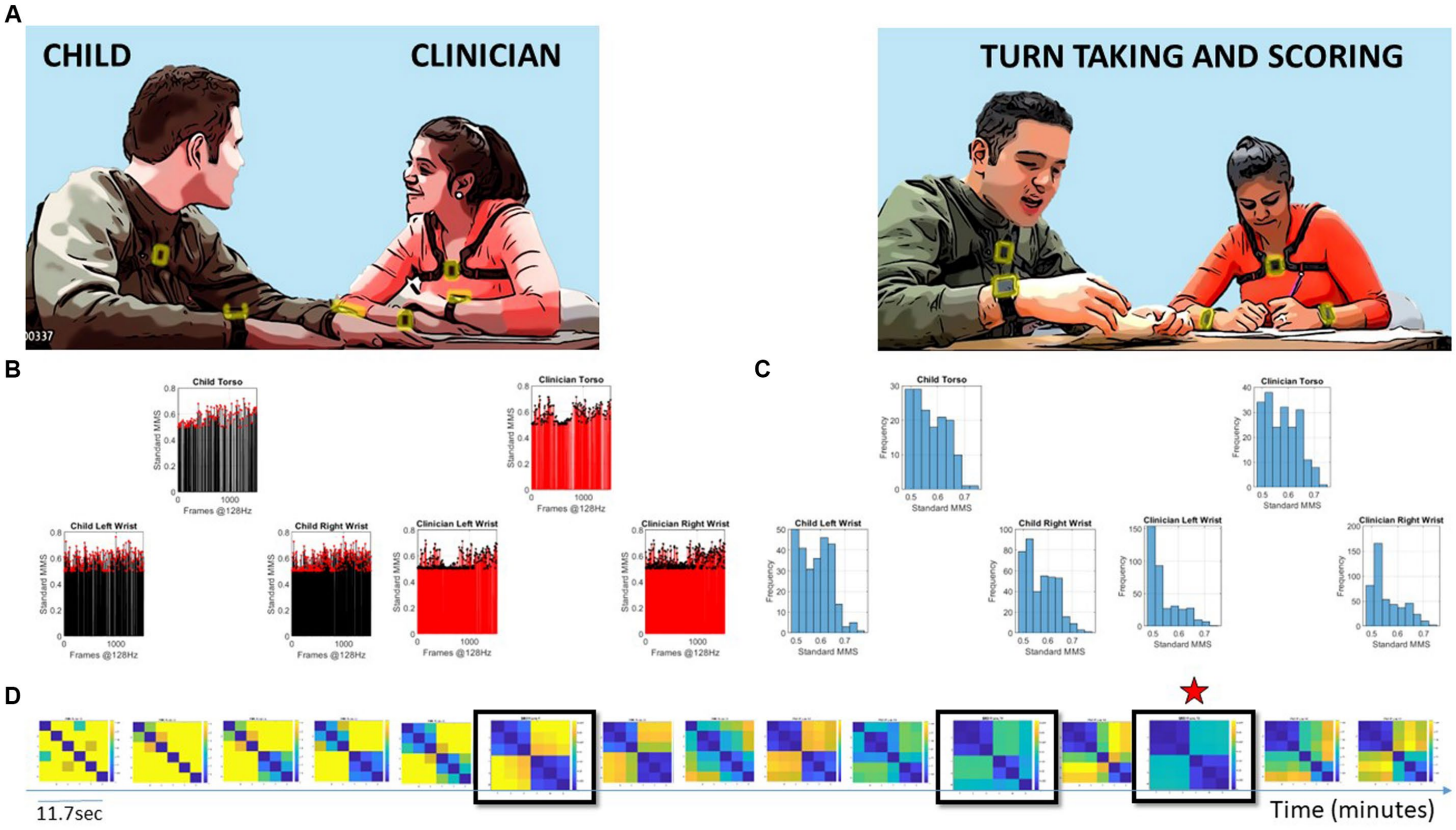
Digitally characterizing socio-motor synergies, rapport, and turn-taking during an ADOS-based social interaction is used here to develop the concept of socio-motor agency through the digital lens. **(A)** Snapshots of the interaction between a child and clinician wearing six biosensors, three on each body, synchronously registering motion at 128 Hz. Clinician-led interaction and note-taking while rating the interaction. **(B)** Sample standardized micro-movement spikes (MMS) derived from angular speed capturing approximately 11.7 s of social exchange. **(C)** Frequency histograms of the MMS peaks (one frame) from each sensor on the child and clinician. **(D)** Pairwise comparison of the histogram evolution using the Earth Movers’ distance similarity metric. Entries reflect the 6 × 6 matrix (child and clinician, three sensors at the torso, right and left wrist of each) as in **(A)**. Off-diagonal entries are the shared dyadic space, while entries next to the diagonal are the child’s or clinician’s activities in standalone mode. Blue-to-yellow color EMD scale ranks from most to least similar spike coincidence patterns. Star marks the maximal similarity.

While in current diagnostic criteria, socially compliant tasks are defined as those that are expected based on some set of rules imposed by some sector of society, in our approach, socially compliant tasks are those that provide a sense of agency to the child that is being diagnosed during a social interaction situation. More precisely, we here define social agency as the balance between autonomy and control during a social exchange. We define autonomy as the ability of the child to lead the conversation as much as the clinician does, rather than always following the lead of the clinician. We define control as the ability of the child to effectively predict the consequences of impending social actions and overall behaviors, based on high levels of motor control. The latter means high signal-to-noise ratio and low randomness in the information being exchanged. Both concepts are illustrated in Figure 1 through the evolution of a dyadic interaction. We further develop the concept in the Methods section of the article.

Socio-motor agency can be impeded if neurodevelopment undergoes a different maturation path (Torres et al., 2013a, 2023; Brincker and Torres, 2013). If the child, for example, has excessive motor noise and motor randomness in its performance, the predictive ability required for self-motor control will be compromised (Torres et al., 2013a), and with it, the overall control ability will be altered. This alteration will also in turn affect the clinician’s perception of the child’s nuanced micro-motions underlying social behaviors, thus biasing the assessment (Torres et al., 2013a; Bokadia et al., 2020; Brincker and Torres, 2013). Indeed, in autism, there is mounting evidence that motor control is fundamentally different from neurotypical development (Torres and Donnellan, 2015; Torres and Whyatt, 2018; Nayate et al., 2005; Fournier et al., 2010), a fact that has been supported across multiple systems. These include the brainstem (Torres et al., 2023), the cerebellum (Frosch et al., 2022; Allen et al., 2004; Sivalingam and Pandian, 2024; D'Mello and Stoodley, 2015), subcortical structures (Chukoskie et al., 2013), the oculomotor system (Ziv et al., 2024), the reaching and grasping system (Torres et al., 2013a), the gait and balance (Vilensky et al., 1981; Accardo and Barrow, 2015; Wu et al., 2024; Hallett et al., 1993; Bermperidis et al., 2021), among others.

Under such circumstances, socio-motor agency can be impeded, as can be the rating of the child by the clinician. Therefore, we propose that the tasks that manifest excess random noise of the joint dyadic motor patterns (lower control of the dyad) and/or excess lead of the clinician within the dyad (lower autonomy of the child) are inevitably bound to bias the clinician’s scoring toward a deficit model of autism. In contrast, the tasks that manifest high dyadic control and autonomy of the child are bound to boost social agency, according to our biometric definition. These tasks can provide a more appropriate model of readiness potential for social exchange.

The current ADOS is a criterion test that has not characterized neurotypical ranges of observed social behaviors. Furthermore, since motor control physiology is not part of the diagnostic criteria of autism, the test says nothing about neuromotor development. However, the physiology of neuromotor development in formative years provides basic building blocks of social–emotional and communication exchange. Here, we characterize normative data on physiological aspects of motor control. To that end, we study the ADOS exchange patterns in neurotypicals of different ages and provide a new characterization of differences in neuromotor development specific to the social communication criteria that the ADOS offers. The new proposed model can also increase the odds of doing such characterization in a fairer, less biased manner. In this sense, the child is offered a chance to succeed at the exchange. In turn, the clinician can presume competence and identify areas of strength to recommend treatments more appropriately. Such treatments would be grounded on the non-obvious, nuanced aspects of behaviors occurring at a micro-level that escape the naked eye of an observer, in addition to the unambiguous aspects of behavior that such inventories use today. These nuances can be quantifiable with biosensors that read out continuous biorhythmic activities from the nervous system with sub-second resolution. At the same time, they would be informed and guided by clinical criteria.

We here introduce a theoretical framework grounded on empirically derived power (scaling) laws of human ontogenetically orderly maturation on a schedule (Torres et al., 2013a). This framework connects stochastic analysis of human biorhythmic (time series) data with information-theoretical metrics. We define new truly personalized computational indexes of dyadic control, autonomy, and socio-motor agency from biosensors’ digital data using as guidance the digitized ADOS-2. Then, we identify socially compliant tasks, i.e., ADOS-2 tasks with balanced socio-motor agency, thus streamlining the digital ADOS-2. Finally, we propose new ways to help automate and speed up autism screening and detection based on these socially appropriate tasks identified from the motor variability of the interactive dyad, complementing those from the child’s or the clinician’s performance alone.

## 2 Methods and analyses

### 2.1 Participants

This study was approved by the Rutgers University Institutional Review Board in compliance with the Helsinki Act. A total of 29 children, including 19 males and 10 females spanning 4–15 years of age, and two adult clinicians participated in the study (see Table 1). Children participated in multiple sessions over the span of 2½ years with one clinician per session and were administered a specific module per session, i.e., a specific subset of ADOS tasks, in accordance with their age, level of development, and spoken language. They were recruited as a random draw of the population, with neurotypicals and autistics signing up for the study through our IRB-approved flyers.

**Table 1:** Participants’ information.

Record number	Participant ID	Age	Sex	Visit 1 module (total score)	Visit 2 module (total score)	Visit 3 module (total score)	Visit 4 module (total score)
1	NT01	8	F	3 (0)	–	–	–
2	NT02	10	F	3 (0)	–	–	–
3	NT03	9	F	3 (2)	–	–	–
4	NT04	12	F	3 (2)	–	–	–
5	NT06	7	M	3 (2)	–	–	–
6	NT08	9	F	3 (0)	–	–	–
7	NT09	7	F	3 (1)	–	–	–
8	NT10	11	M	3 (1)	–	–	–
9	NT11	15	M	4 (2)	–	–	–
10	NT12	11	F	4 (1)	–	–	–
11	NT13	13	M	4 (0)	–	–	–
12	EP01	4	M	3 (10)	2 (7)	3 (9)	2 (8) *X
13	EP02	8	M	3 (9)	2 (8) *X	–	–
14	EP03	10	M	3 (12)	2 (13)	3 (24) *X	2 (21) *X
15	EP04	13	F	3 (7)	4 (8) *X	3 (11) *X	4 (8) *X
16	EP05	6	M	3 (9)	2 (7)	3 (8) *X	–
17	EP07	11	M	3 (12)	2 (9) *X	3 (18) *X	2 (13) *X
18	EP09	5	M	1 (18) *X	1 (16)	–	–
19	EP10	9	M	1 (13)	1 (17)	–	–
20	EP13	6	M	3 (17)	–	–	–
21	EP14	14	M	1 (8)	2 (10)	1 (14)	2 (15) *X
22	EP15	10	M	1 (17)	1 (15) *X	1 (26)	1 (21) *X
23	EP16	4	M	3 (11)	2 (11)	3 (22) *X	2 (20) *X
24	EP17	11	M	1 (16)	1 (18) *X	1 (18)	1 (19) *X
25	EP18	9	F	3 (10)	2 (11)	3 (16)	2 (10)
26	EP19	7	M	3 (8) *X	2 (8) *X	3 (8)	–
27	EP20	7	M	3 (11)	2 (11) *X	3 (17) *X	2 (16) *X
28	EP21	11	F	3 (11)	2 (9) *X	3 (8) *X	2 (11) *11
29	EP22	4	M	1 (26)	1 (24) *X	–	–

*X denotes that the participant came for the visit, but his/her data could not be used either because it was not available/lost/corrupted or the information available was incomplete. NT stands for neurotypical participant, and EP stands for expected ASD participant. The latter were confirmed by the clinician to have an ASD diagnosis at the end of the session.

### 2.2 Raw data acquisition

Digital data were acquired during each session using light wearable sensors (APDM Opals, Portland, OR, United States). Six sensors were used, two on the left and right wrist and one on the torso, both on the child and clinician. The sensors continuously and synchronously recorded triaxial accelerometry and gyroscopic data at a sampling frequency of 128 Hz. The recording environment followed the standardized ADOS requirements using similar table and sitting arrangements for the clinician–child dyad. The two clinicians were unaware of the goals of the study.

### 2.3 Data type: the micro-movement spikes derivation

The micro-movement spikes (MMS) are a data type that we invented (Torres et al., 2013a) to create a standardized time series representing the moment-to-moment fluctuations in signal amplitude and timing relative to an empirically estimated mean while scaling out allometric effects. Allometric effects are due to anatomical disparities across participants (Lleonart et al., 2000). Anatomical disparities influence the kinematic parameters, and as such, confound the results from the analyses (Torres et al., 2018). The MMS can be obtained from any biorhythmic activity registered with biosensors or cameras, consisting of peaks and valleys changing over time. For example, they can be time series of triaxial acceleration from accelerometers (inertial measurement units) or from triaxial angular velocity obtained with gyroscopes. In the present study, we focus on the latter. Scalar values of angular speed from orientation data that the gyroscopes recorded were acquired using the Euclidean norm (Equation 1) of the coordinate components of motion as measured by the sensors:


(1) $$V=\sqrt{V_{x}^{2}+V_{y}^{2}+V_{z}^{2}}$$

From here onward, all analyses refer to the scalar value V, the angular speed in deg/s.

To scale out allometric effects, we normalize motion data fluctuations (peaks and valleys) (using Equation 2) as relative deviations from the empirically estimated mean activity of the series. To that end, we use maximum likelihood estimation (MLE), and upon testing various families, we obtained the shape and scale parameters of the continuous Gamma family of probability distribution functions. From these empirically estimated parameters, we obtain the moments and use the Gamma mean to shift and center our data around the estimated mean. Then, we obtain the local absolute deviations from the empirically estimated Gamma mean. We scale the mean-relative deviations to the [0,1] interval according to the local minima average:


(2) $$\mathrm{NormPeak}=\frac{\mathrm{Peak}\;}{\mathrm{Peak}+Avg_{\min to\;\min}}$$

Here, 0 values represent 0-deviations from the empirical mean of the session. They are “quiet moments” relative to the person’s mean. They are the baseline value of the individual and provide a personalized signature that changes over time or with the context. For this reason, we focus on the relative deviations from this individual’s baseline. Values away from 0 value represent activity above the person’s baseline mean within the given situation. The normalized peak series of MMS (Torres et al., 2013a; Wu et al., 2018) conserve the temporal structure of the original speed/acceleration time series but are normalized (Torres et al., 2018). This normalization provides standard ranges for each person and permits comparison of motor biorhythmic patterns across individuals, independent of their age differences and corresponding disparate anatomical lengths. Supplementary Figure S1 provides the pipeline to compute the MMS.

### 2.4 The gamma process of the MMS

The normalized angular speed MMS is best fit (in the MLE sense) by the continuous Gamma family of probability distributions (Torres et al., 2013a; Torres, 2011a). Furthermore, the parameters of the Gamma distribution, shape k, and scale *θ* have been found across multiple studies from our laboratory, including the present one (see Results), to follow a scaling power law of the form described in Equation 3:


(3) $$k≅a\theta^{b}\to \log\left(k\right)=a+\mathrm{blog}\left(\theta \right)+\varepsilon$$

where *ε* is a small error term and *b* < 0. This power law for the standardized MMS time series reveals a maturation process of the motor code for voluntary (Torres et al., 2013a, 2016a) and involuntary (Torres et al., 2020a) motions. This law is very important because it provides us with a quantitative framework to interpret fluctuations in biorhythmic data that range from random to predictive. Furthermore, they offer a form of ground truth to quantify deviations across the human lifespan. Because of this tight linear relation of the log scale and log shape, knowing one helps us infer the other with high certainty.

Importantly, the continuous Gamma family of probability distributions has the first (mean) and second (variance) moments expressed in terms of the shape and scale described by Equation 4).


(4) $$\mu =k\theta ,\sigma^{2}=k\theta^{2}$$

Then, using Equation 4, the noise-to-signal ratio (NSR) of the MMS reduces to the Gamma scale parameter as in Equation 5:


(5) $$NSR=\frac{\sigma^{2}}{\mu }=\frac{k\theta^{2}}{k\theta }=\theta$$

The Gamma scale parameter in Equation 5 fully characterizes the noise of the motor patterns of the interactive dyad (or of the participant), i.e., in relation to the level of fluctuations of angular speed during the ADOS activities.

Empirical estimation of these parameters in thousands of participants over more than a decade of work involving humans along the lifespan, and across disorders of the nervous system, has revealed an interpretation for the Gamma log–log parameter plane. Distributions that fall along high NSR regimes are also close to the memoryless random regime of the special exponential distribution case (when k = 1). Points in mid-NSR correspond to heavy-tailed Gamma distributions. Then, low NSR (or high signal = 1/NSR) are congruent with symmetric shapes (Gaussian-like) distributions. High-signal Gaussian regimens are highly predictable in contrast to high-noise memoryless random exponential regimes. As such, this parameter plane is empirically interpretable. Supplementary Figure S2 shows the Gamma process interpretable parameter spaces.

### 2.5 Quantifying motor control from the perspective of an agent

Noise-to-signal ratio measures the degree of motion variability away from mean activity. Low regimes of NSR characterize steady and smooth motion, akin to goal-oriented behavior, as experienced from the perspective of the agent/child. On the other hand, high NSR regimes indicate unpredictable and random motion. This is so because of the scaling power law, since knowing one parameter infers the other. At high NSR, the tendency of the distributions is toward the memoryless exponential, representing randomness. In that sense, the high NSR is a proxy for low motor control. In contrast, high signal (or low NSR) infers the existence of predictable motor patterns toward the Gaussian regimes of the Gamma process. Because the NSR is calculated on the standardized MMS, motor noise does not depend on the anatomy of the individual as it is scaled by the mean amplitude of motion. This enables us to plot all participants across different ages on the same parameter space.

### 2.6 An information theoretic approach to the analyses of the MMS

The presence of MMS peaks indicates an outburst of activity away from the individual’s baseline. This is informative of motor activity within a given context, which in this case was maintained similarly across participants, as per ADOS requirements. When we also consider the temporal distribution of MMS peaks, a train of such spikes can be viewed as a representation of information regarding human motion variability through time. Considering multiple sensors sampling in synchrony, the MMS spikes carry spatiotemporal information about the bursts of distributed bodily activity in the person’s motor system.

Following our argument, we redefine socio-motor agency as the balance between motor control and motor autonomy, using these physiologically grounded biosensor data. Signal-to-noise ratio characterizes the ability of an agent to (internally) control their own motor behavior. Entropy rate characterizes the ability of the agent to motorically act autonomously (while minimizing external control by another agent) in a social interaction.

**Figure 2:**
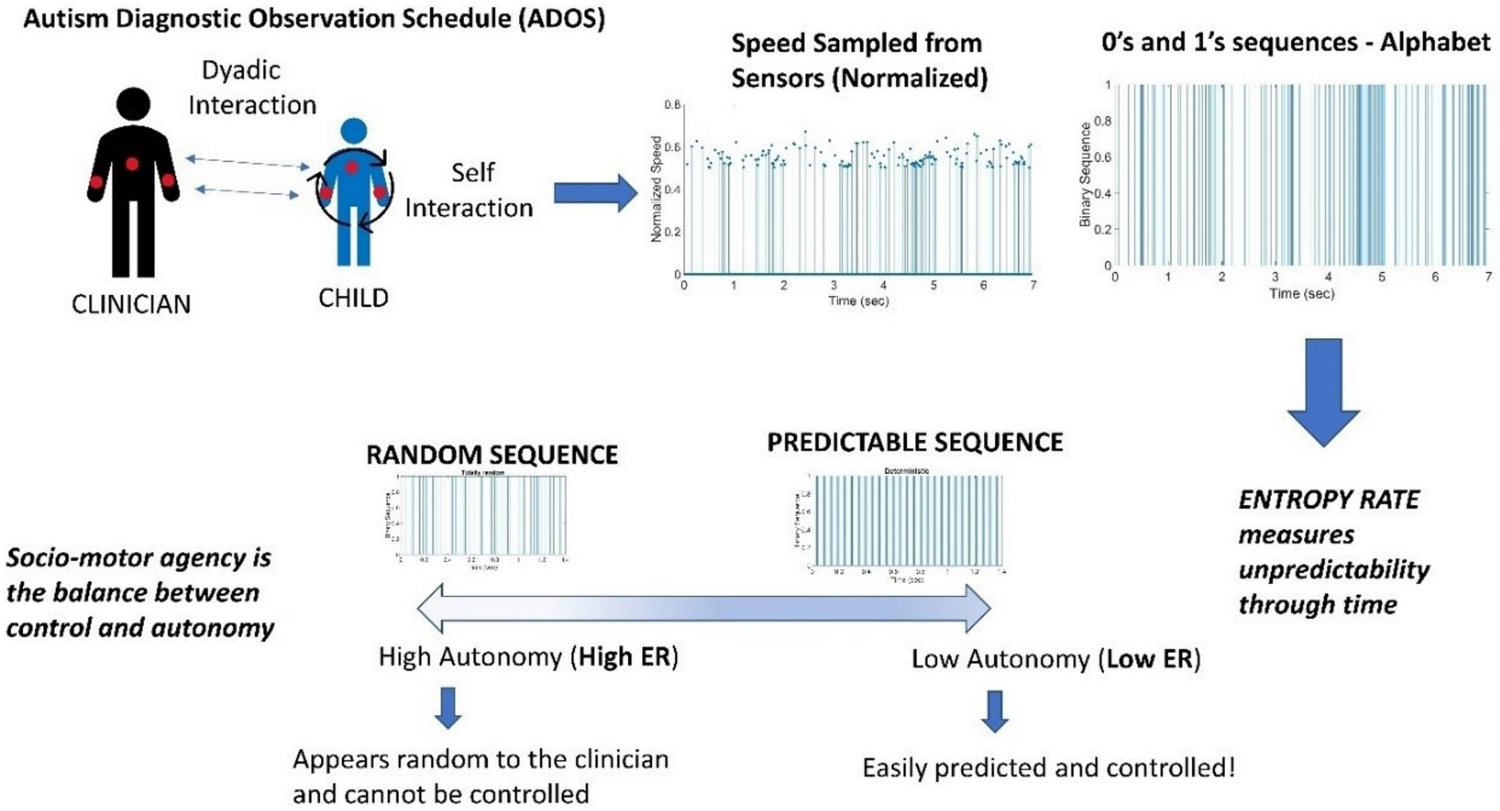
Digitization of the Autism Diagnostic Observation Schedule (ADOS): Angular speed samples (128 Hz) from wearable sensors on the wrists and torso of the child and clinician are normalized and binarized to obtain discrete sequences of 0’s and 1’s. Entropy rate estimates measure the unpredictability of the underlying binary processes to characterize the agents’ autonomy in the dyadic social interaction. The analysis is performed on data from time windows of ~7.8 s, which proved optimal to attain tight 95% confidence intervals.

Finally, using transfer entropy, we can quantify the amount of causal influence from the clinician to the child and vice versa, without the need to use any model or make any other assumptions. Figure 2 depicts these proposed concepts in schematic form.

The main concepts and formulas that we introduced to characterize control and autonomy can be summarized in Table 2, and we refer the reader to the Supplementary material where we further expand on these concepts and define the clinically relevant parameters as well.

**Table 2:** Glossary of main concepts, formulas, and their respective domains.

Concept	Equation	Range
**Control**	$\frac{1}{NSR}$ , $NSR=\frac{\sigma^{2}}{\mu }=\frac{k\theta^{2}}{k\theta }=\theta$ Micro-movement sequence	$\left(0,+\infty \right)$ **Memoryless exponential, low signal to predictive, high signal Gaussian**
Entropy	$H=-\underset{X_{1},X_{2},\ldots X_{N},}{\sum }P_{X_{1},X_{2},\ldots ,X_{N}}\log_{a}\left(P_{X_{1},X_{2},\ldots ,X_{N}}\right)$	$\left(0,+\infty \right)$
Entropy rate of a signal (ER)	$H\left(X\right)=\frac{1}{T}\lim_{T\to \infty }H\left(X_{1},X_{2},\ldots ,X_{T}\right)$	$\left(0,+\infty \right)$ : continuous (0, $\log_{a}K$ ): K symbols, base α
ER approximation	Approximate entropy $\mathrm{ApEn}\left(m,r,N\right)$ $≅\frac{1}{Ν-m}\overset{N-m}{\underset{i=1}{\sum }}\log_{a}\frac{\sum_{j=1}^{N-m}\left[\#j,d\left[\left|x_{m+1}\left(j\right)-x_{m+1}\left(i\right)\right|\right]<r\right]}{\sum_{j=1}^{N-m}\left[\#j,d\left[\left|x_{m}\left(j\right)-x_{m}\left(i\right)\right|\right]<r\right]}$	$\left(0,+\infty \right)$ : continuous (0, $\log_{a}K$ ): K symbols, base *α*
**Autonomy**	Entropy rate of micro-movement sequence	
**Socio-motor agency**	**(Control, Autonomy)**	
Local conditional mutual information	$i\left(x;y|z\right)=\log_{2}\frac{p\left(x|y,z\right)}{p\left(x|z\right)}$	$\left(0,+\infty \right)$
Local transfer entropy	$t_{Y\to X}\left(n+1,k,l\right)=i\left(y_{n}^{\left(l\right)};|x_{n+1};|x_{n}^{\left(k\right)}\right)$	$\left(0,+\infty \right)$
Transfer entropy	$T_{Y\to X}\left(k,l\right)=E\left[t_{Y\to X}\left(n+1,k,l\right)\right]$	$\left(0,+\infty \right)$

Novel definitions are shown in bold.

For more in-depth details on information theoretical concepts, formulations, and models that were used in this study, please consult Chapter 1 of the Supplementary material.

## 3 Results and discussion

### 3.1 Age-dependent dyadic motor control separates neurotypical (NT) from children with autism spectrum disorders (ASD)

**Figure 3:**
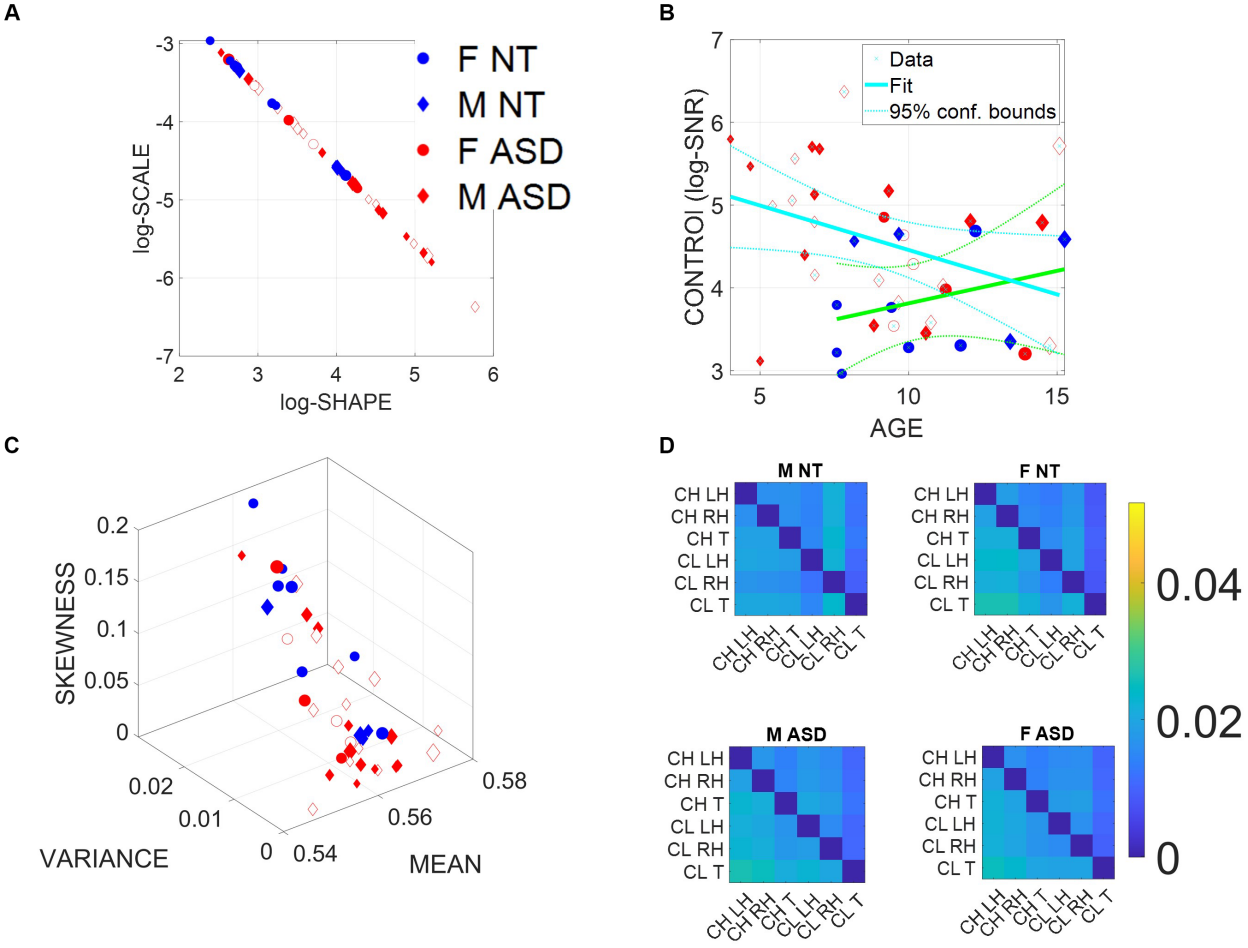
Summary of stochastic characterization of micro-movement spikes, MMS, derived from ADOS-driven dyadic interactions, using angular speed registered from the right (dominant) wrist. Activity encompasses the entire ADOS session. Filled markers represent first visits to the clinician; unfilled markers are subsequent visits. **(A)** Empirically estimated Gamma Shape and Scale (NSR) for each participant using Modules 1, 3, and 4 of the ADOS test as a backdrop behavioral assay. The size of the marker is proportional to the age of the participants. Empirical Gamma plane of individual children and clinicians separating young from older children and adults in an interpretable map of human neuromotor maturation. **(B)** Child’s negative Gamma scale parameter (−log NSR = log SNR) denotes control as a function of age. Observe (cyan line) that after school age, there is a decreasing trend of SNR with age for ASD in contrast to the opposite trend for NT (green line). **(C)** Parameter space spanned by the empirically estimated Gamma mean (*x***-**axis), standard deviation (*y*-axis), and skewness (*z*-axis) derived in **(A)**. Marker size is proportional to age. **(D)** Quantification of transfer entropy for social dyads involving clinician and child obtained for males and females in the NT and the ASD groups, using six sensors, three on the clinician and three on the child, outputting time series of angular speed motion on the left and right wrists and the trunk of each social agent in the dyad. Off-diagonal entries represent joint dyadic activities.

The empirically estimated Gamma parameters localized each child–clinician’s dyadic interaction for each task on the Gamma parameter plane with 95% confidence intervals (Figure 3A). These points represent the empirical probability density function (PDF) of their joint dyadic interaction. When we plot the full scatter estimated from each task in the ADOS for all children, a tight linear relation emerged whereby the log–log plot follows a scaling power law of the form $k≅a\theta^{b}$ . [see Methods for a more in-depth analysis of the power law and the micro-movement spikes (MMS) time series transformation].

This relationship, first described as a maturation law in humans’ voluntary decision-making, mediated by pointing motions (Torres et al., 2013a), is reproduced here for gyroscopic data reflecting joint dyadic angular speed, such that as the Gamma scale value decreases, the Gamma shape value increases. Because knowing one, we can predict the other with high certainty, we can then reduce these two parameters of interest to one parameter summarizing these motor signatures of the interacting dyad. We can also do so for each individual signature, i.e., those of the child and those of the clinician in standalone mode.

Importantly, the continuous Gamma family of probability distributions has the first (mean) and second (variance) moments expressed in terms of the shape and scale as in Equations 4, 5:

The Gamma scale parameter in Equation 5 fully characterizes the noise of the biorhythmic motor patterns of the interactive dyad, i.e., in relation to their joint level of fluctuations of angular speed during the ADOS activities.

Prior research from our lab concerning autonomic (Elsayed and Torres, 2023), involuntary (Torres and Denisova, 2016), automatic (Bermperidis et al., 2021), and voluntary movements (Torres et al., 2013a; Wu et al., 2018) and research from other labs involving the oculomotor systems (Ziv et al., 2024) has consistently shown across levels of functionalities and systems that high noise levels and randomness correlate with levels of autism severity. Specifically, high-signal Gaussian regimens are highly predictable in contrast to high-noise memoryless random Exponential regimes.

The Gamma parameter plane, which is empirically interpretable, provides criteria for the locations of the Gamma distribution shape and scale parameters. They characterize the signatures of individual participants and change in an ontogenetically orderly manner whereby a decrease in the NSR is accompanied by a decrease in randomness (away from the memoryless exponential distribution at shape = 1), and this decrease, in turn, corresponds to human neurodevelopmental maturation.

The stunting of maturation of the human nervous system has then been well characterized by high NSR and random fluctuations previously found across different ages in ASD (Torres et al., 2013a). In this sense, we equate high SNR = 1/NSR with an index of controllability. As per the scaling power law, high SNR of the MMS is equated with high predictability of the person’s self-referenced, self-generated motor code. Our lab has proposed that this motor code represents a proxy of kinesthetic reafference, i.e., the continuous stream of re-entrant motor activity from the periphery, serving as an index reflecting the quality of the motor feedback to the central controller of the nervous system (Torres et al., 2013a; Brincker and Torres, 2013).

Then, as this motor code is shared with another agent during social dyadic interactions, the distributions of the joint dyadic interactions of the participant and the clinician for the 26 participants (11 neurotypically developing NT and 15 ASD) can be appreciated in Figure 3A following a power law. These distributions are derived from the MMS that fluctuations in angular speed produced in the dominant hand (see Methods; Figure 2).

Furthermore, Figure 3B shows that the log (SNR) = −log(NSR) (denoted as the index of control) of the interacting socio-motor dyad has an age-dependent pattern. In NT children, as the age increases, control tends to slightly increase, i.e., a slight positive trend is reflected in the slope of the line fitting the (blue NT scatter), NT: intercept = 3.0271 *p* = 0.0067, slope = 0.0786, *p* = 0.362. In contrast, as ASD children age, control tends to decrease, i.e., a strong negative trend is quantified in the slope of the line best fitting the red ASD scatter: intercept = 5.5317, *p* = 5.65 × 10^−12^, slope = −0.1074, *p* = 0.0463.

In Figure 3C, we compare the two groups by localizing each participant on the Gamma moments space spanned by the empirical mean, variance, and skewness, whereby each point represents the empirically estimated moments of the Gamma PDF of joint dyadic activity for each child–clinician pair. This result demonstrates a tendency of the joint dyad moments to separate NTs from ASD participants, as they interact with an adult clinician, expressing marked differences between males and females.

To better appreciate the sex differences, we obtain pairwise the transfer entropy (TE) from child to clinician and from clinician to child. See Methods for an extended definition, but recall that TE is the reduction in uncertainty of predicting the future of X when we consider the process Y. In Figure 3D, we can see for each matrix the pattern that emerges when considering the time series data from each of the six sensors attached to the child’s and clinician’s two hands and trunk. The cross terms in the off-diagonal entries of the matrix (top right-hand entries 1,4 to 1,6; 2,4 to 2,6; 3,4 to 3,6; and bottom left-hand entries 4,1 to 6,1; 4,2 to 6,2; and 4,3 to 6,3) represent the dyadic cases of child to clinician and clinician to child, respectively. In the shared entries of the matrix, we see that in ASD, males show a decrease in TE values while females show an increase. In the context of the ADOS, females evoke a reduction in the clinician’s uncertainty predicting the impending females’ motions, i.e., perhaps an inherent bias that partly accounts for the disparate ratio of approximately 4–5 males per female diagnosed with ASD (D'Mello et al., 2022). We will further explore these differences to try and understand the interplay between the NSR as an index of motor controllability (predictability) and the overall sense of socio-motor agency in each of the ADOS tasks, for males and females.

In the diagonal sub-matrices (top left-hand entries 1,1 to 1,3; 2,1 to 2,3; 3,1 to 3,3; and bottom right-hand entries 4,4 to 4,6; 5,4 to 5,6; and 6,4 to 6,6), we represent the patterns within the individual’s body parts. There we appreciate higher values of TE from child to child in both ASD males and females, with ASD females having higher TE than ASD males. As with the shared dyadic activity, here in the individual patterns, the highest differences for clinician to clinician can be appreciated in the ASD females.

### 3.2 Quantifying dyadic social agency reveals differences between NT and ASD

High levels of NSR in the MMS fluctuations from the angular speed coincide with memoryless random regimes of motor patterns—well characterized by the exponential distribution previously found in autistic individuals (Torres et al., 2013a; Torres, 2011b). It has been proposed that under such random and noisy motor code, it is difficult to have high-quality motor feedback contributing to a predictive code. Such predictive code would be necessary to compensate for sensory-motor and inertial time delays inherent in the nervous system (Torres et al., 2013a; Brincker and Torres, 2013, 2018; Torres, 2011b; Mohamed Thangal and Donelan, 2020).

In a dynamic dyadic social interaction such as that taking place during the ADOS, it is then difficult to exert control over the interaction because presses by the clinician and overtures by the child are not occurring at the expected timely rates. This temporal mismatch in autism alone can bias the rating by the clinician in ways that differ between NT and ASD but also may differ between males and females. Here, we equate high NSR with low predictive control and posit that the type of socio-motor agency required in a naturalistic social interaction will be impacted by poor controllability levels on one side of the dyad. We then question whether dyadic-based control (i.e., shared by the child and clinician) is differentially impacted in ASD participants.

Another aspect of dyadic social agency is motor autonomy. As mentioned earlier, motor autonomy is defined here as the ability of the child to lead the conversation as much as the clinician does, rather than always follow the lead of the clinician. An obvious way to quantify the degree to which the clinician is leading the exchange would be by using some form of causal analysis between data recorded on the clinician and data recorded on the child. As our main approach, however, we choose to quantify autonomy in ways that depend on data recorded from wearable sensors on a single agent which as we will show, is intuitive and can be applied in a clinical setting, to help digitize the ADOS.

We introduce (behavioral) spike trains from the MMS derived from the time series of angular speed. We use entropy metrics to examine the degree to which the spikes behave randomly or deterministically (i.e., containing periodic, systematic patterns). To that end, we use entropy rate, a metric well suited to interrogate the stochastic regimes of spike trains (Delgado-Bonal and Marshak, 2019; Lizier, 2014).

From the MMS derived from time series of angular speed, recorded either from the left or the right hand of the child, we derive binary sequences whereby a sequence of 1’s corresponds to bouts of activity and 0’s to “quiet” sampling periods, when no significant change above the person’s average activity occurs in the angular speed profile. Another way to view these binary sequences is as the manifestation of an underlying “alphabet” that characterizes the predictability of the motor code. Zeros and ones will appear with some probability, which we expect to change at some time scale due to the non-stationary nature of human motion. But if we restrict ourselves to small time windows (~7.8 s, determined as optimal for empirically estimated confidence intervals, upon sampling different sizes), this time window is small enough that the process can be viewed as stationary yet large enough to contain a satisfactory number of samples, lending statistical power to our empirical estimation per window. As such, 7.8 s is our unit of time for the spike trains that we derived. Using this MMS per unit of time as our data type, we can then measure the degree of randomness of the child’s motions by estimating the entropy rate (see Methods). Furthermore, we then compare it to transfer entropy (TE) obtained from the child and clinician, a causal metric that can inform us of who leads the interaction for any given task.

We argue that a suitable scale of motor autonomy is one in which, at one extreme, a high degree of randomness is a measure of a system at its highest degree of motor autonomy. This is the type of state where the system is uncontrollably “hidden” from the controller. There is no opportunity to control the person. At the other end, the lowest degree of randomness leads to a systematic, deterministic pattern, highly controllable. While in the former, the child’s system with excessive autonomy prevents social exchange with the clinician in that the clinician cannot control the child, in the latter, the clinician can absolutely control the child. Either extreme is detrimental to the development of rapport or turn-taking in a social exchange. A happy medium is one in which, while the child preserves a degree of autonomy that enables a balanced social exchange, the clinician also partakes in a give-and-take interaction rather than leading the child most of the time.

**Figure 4:**
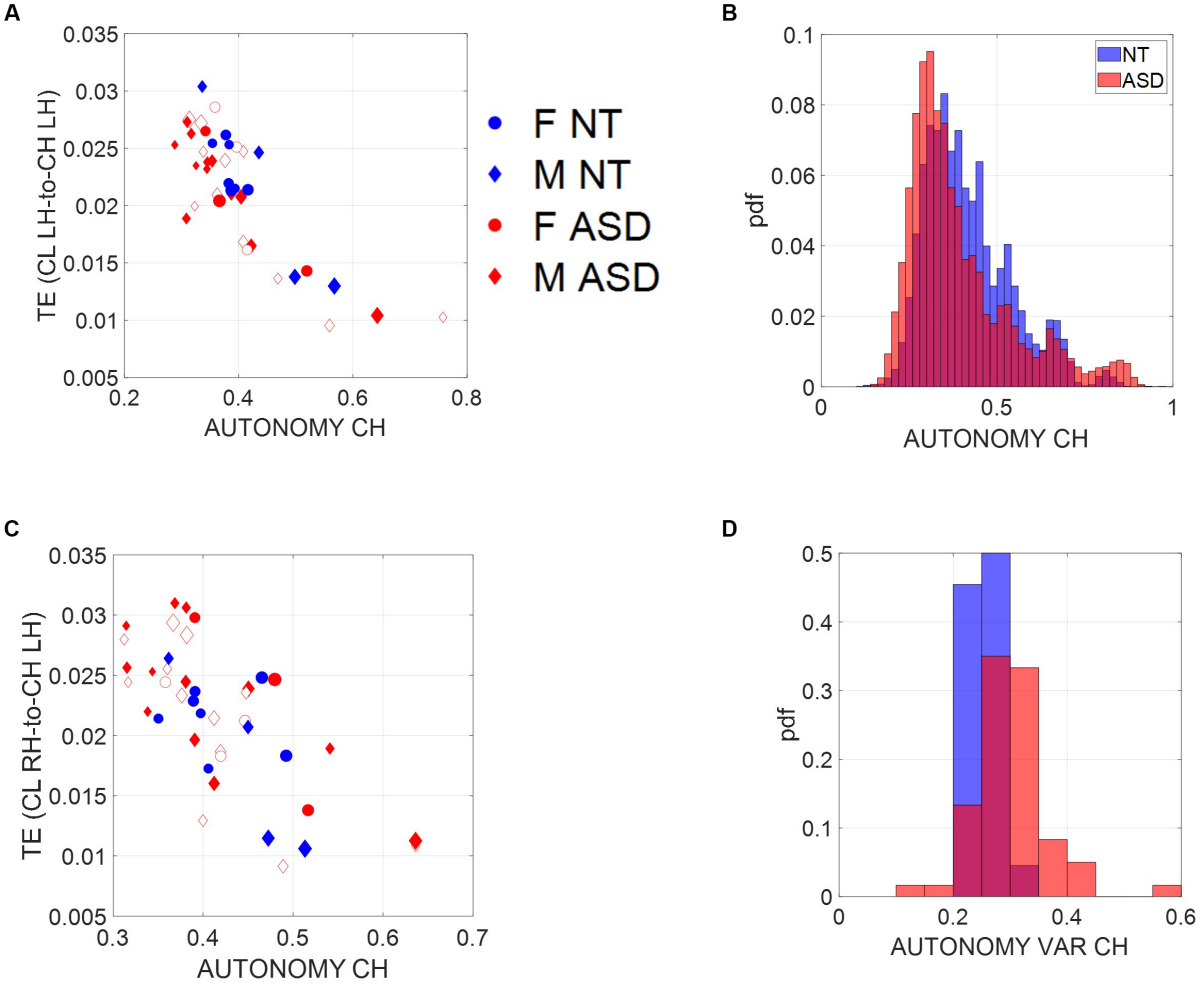
Scales of socio-motor agency according to index of autonomy. **(A)** Average transfer entropies between child and clinician taken over windows of ~7.8-s duration, per participant (filled-in markers are first visit to the clinician, unfilled markers are subsequent visits) versus autonomy (Ap Ent) reveal higher autonomy index in NT, a trend that is also quantified in **(B)**. As the child’s autonomy decreases, the CL to CH TE (measured in left hand) increases. Adding the CL past activity does not contribute more information about the CH state than looking at the CH past activity alone. **(C)** This is also the case for the right hand. **(D)** Autonomy variability (variance over the mean) throughout a session is higher for the ASD group, including both the child and the clinician involved.

We test our new hypothesis that motor autonomy relates to measures of entropy by comparing TE (a measure of causality) from the child to the clinician with entropy rate, a measure spanning a scale from totally random to totally deterministic states of the spike-based code. We show in Figure 4A an age-dependent trend spanning two scatters. In older neurotypical children, the scatter aligns such that as the child’s entropy rate (denoting our scale of motor autonomy) increases, so does the TE, denoting a causal lead of the child over the clinician. In contrast, a second scatter emerges for younger children, whereby the trend is less visible, indicating that these children’s index of motor autonomy is not as evident during the exchange and the causal lead (TE), denoting the child’s lead over the clinician’s lead, is less evident. We can appreciate the shift in this metric of motor autonomy in Figure 4B, where the histogram of the ASD children is shifted to the left, indicating lower density values than NT children.

Since the left hand is not the dominant hand in these children, we plotted the histograms pertaining to the left hand as well (Figure 4A, left hand and Figure 4C, right hand) to see if these effects consistently emerged. We see in Figure 4B that across multiple time windows, the pdf for the neurotypical group is shifted to the right, meaning that on average, NTs have higher values of autonomy than ASDs. Since autonomy also varies throughout sessions, plotting the autonomy variability (variance of this index over the mean of this index) for different participants in Figure 4D shows that for the ASD group, child and clinician variability is higher than most NTs. This variability index tends to separate ASD from NT participants, particularly for later visits (as the child aged, over 2 years and a half that the study spanned).

### 3.3 Age-dependent motor autonomy across children versus clinician’s motor autonomy robustness

As we saw earlier, the SNR (1/NSR) of the control index has a trend with age that differs between the two groups. NT children show increasing control with age, whereas ASD children show a decreasing trend. Similarly, here we ask if the index of motor autonomy also changes with age. To that end, we examine this index as a function of age across the children. We also examine it for the mature adult clinician across the children’s ages.

**Figure 5:**
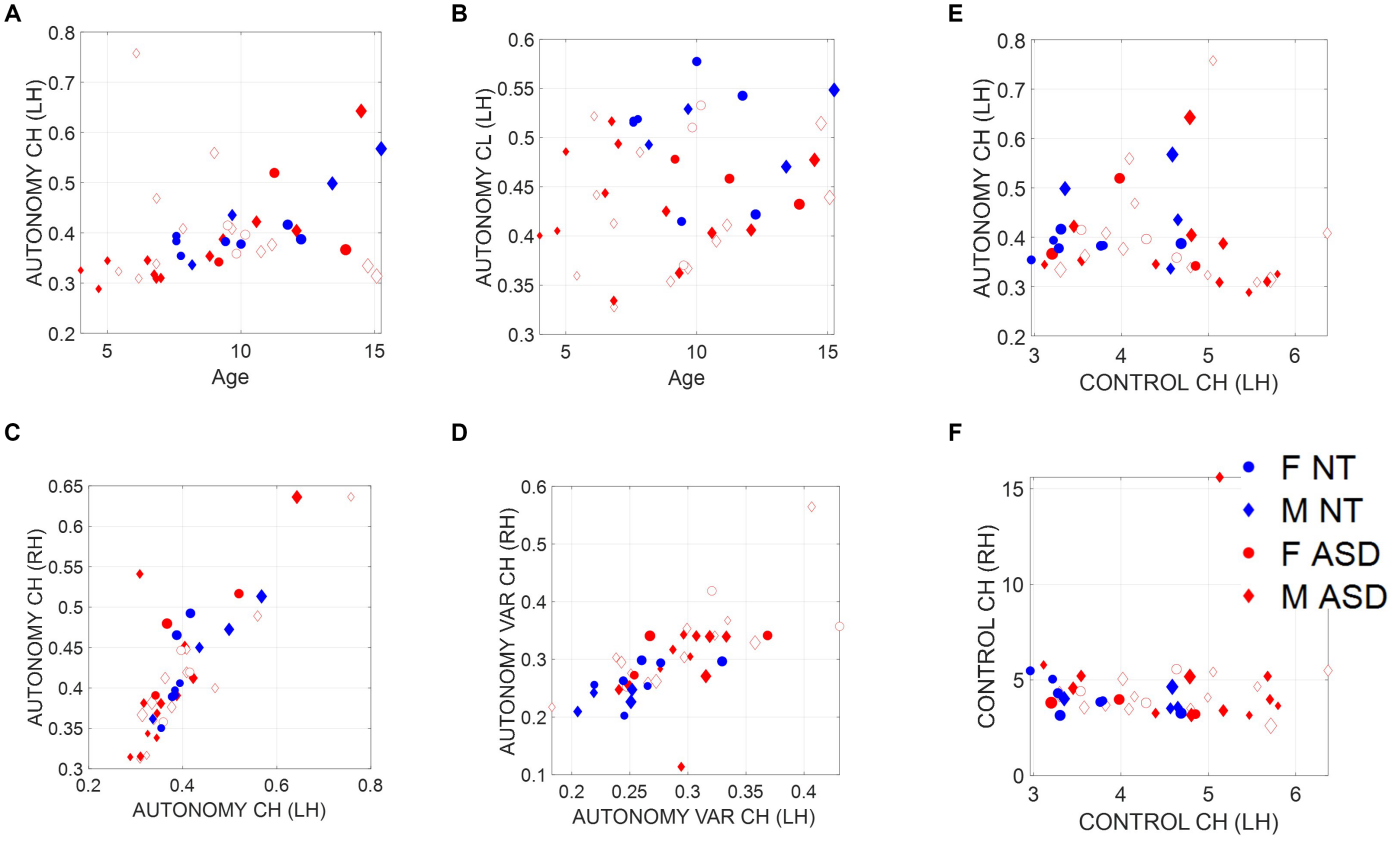
Non-equivalence of the index of motor autonomy and the index of motor control. Plots reflect the average child and clinician index of motor autonomy for left-hand motions versus age as well as right-versus left-hand index of motor autonomy, index of motor autonomy variability, and index of motor control. **(A)** Child index of motor autonomy is positively and linearly correlated with age. **(B)** There is no trend between the clinician index of motor autonomy across children’s ages. **(C)** Equivalence of index of motor autonomy derived from the left hand versus the right-hand motions. **(D)** Index of motor autonomy variability also correlates between the two hands and separates NT (blue) versus ASD (red). **(E)** No definite relationship between index of motor control and index of motor autonomy is observed; however, for small values of motor control index there seems to be a positive trend with autonomy, which then becomes negative for high values of motor control. **(F)** Left-hand motions have higher variability in the index of motor control than do right-hand motions. Notice the independence from **(C)**, representing autonomy.

We find that the child’s index of autonomy for both NT and ASD increases with age in all cases (Figure 5A). This result reveals that the ability of the ASD child to actively participate in a dyadic interaction is a human socio-motor developmental trait that improves with age. In contrast, Figure 5B shows that the clinician’s autonomy is independent of the child’s age. In this case, the adult clinician shows no discernable trend.

### 3.4 Indexes of autonomy and control are not equivalent

The child’s index of motor autonomy and the variability of this index extracted from the sensors in both hands are linearly correlated (Figure 5C). In the case of the index of motor control, however, there is higher variability of the mean motor autonomy index across subjects when we use data from the left-hand sensor (as shown in Figure 5D, where separation of the NT from ASD is evident). For this reason, we focused our analysis on the non-dominant, left-hand motions. Furthermore, the index of motor autonomy derived from the left-hand motions as well as its index of motor control are positively correlated for small values of motor control index but negatively correlated for higher values. This is shown in Figure 5E. In other words, autonomy and control are not equivalent metrics. This can be further appreciated in Figure 5D (motor autonomy variability) versus Figure 5F (index of motor control).

### 3.5 Male versus females respond differently to ADOS tasks—the case of ASD females

In addition to the quantification of *indexes of* motor control and motor autonomy as components of socio-motor agency, we rendered it important to consider the heterogeneity of tasks in the ADOS’ modules 1, 3, and 4 used here across children with different levels of spoken language. We grouped tasks into three main categories: Socio-Motor, requiring high motoric components (frequent movements and gestures); Abstract, tasks more “mental” in nature, requiring abstraction, theory of mind, and other cognitive components; Emotional, tasks that elicit feelings and emotional reactions, strongly *visibly* impacting the child’s emotional states.

We calculated the average indexes of motor autonomy and motor control across all participants, derived from samples corresponding to the different ADOS tasks. Then, we assessed potential differences between ASD and NT participants, focusing on the comparison of males versus females. We found that ASD males respond with lower index of motor autonomy than do NT males. In contrast, ASD females versus NT females, manifest very modest differences, inclusive of three tasks with no significant differences [Social Difficulties and Annoyance, Loneliness (both Emotional type tasks), and Construction Task (Socio-motor type task)].

**Figure 6:**
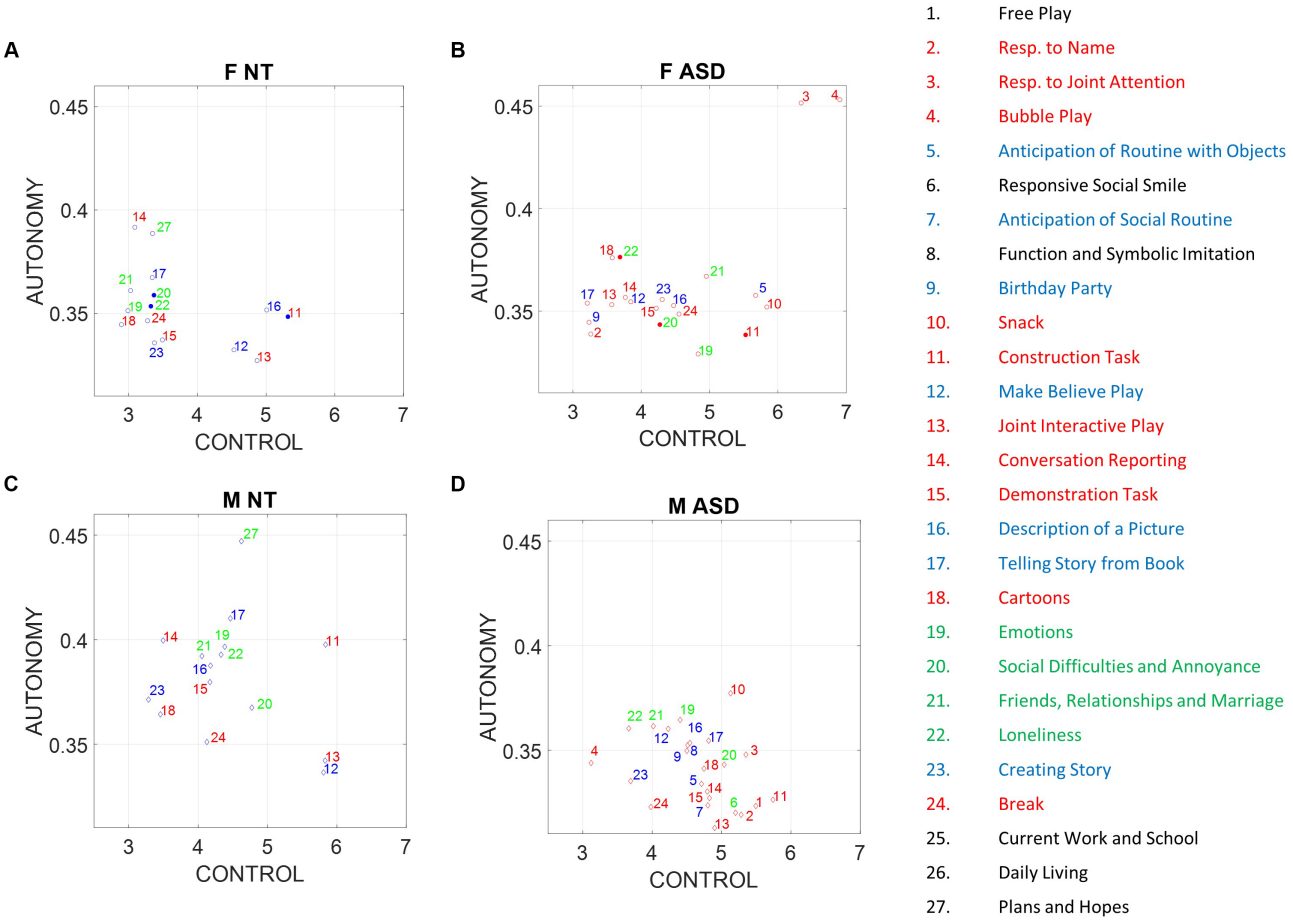
Differences between males and females in average index of autonomy versus control. Filled circles code non-significant differences at the 0.05 level, while non-filled circles denote significant differences between NT and ASD participants. **(A)** NT females. **(B)** ASD females. **(C)** NT males. **(D)** ASD males.

It is therefore clear that ADOS tasks inherently bear a lack of differentiation between NT and ASD females, unlike their male counterparts, for which the differences are large. This can be appreciated in Figures 6A,B (females) and Figures 6C,D (males), where we color code the task type and code it numerically according to the name of the task (the Methods section describes the ADOS tasks included from each module). Modest differences were observed between females.

Notice that despite the non-significance in differences of emotional and socio-motor tasks, emotional tasks have broader spread in ASD females along the index of motor control than do NT females. In contrast, the index of motor autonomy has comparable spread for both. As socio-motor agency is defined as the ratio of index of motor autonomy/index of motor control, this implies that across these ADOS emotional tasks, NT females have more socio-motor agency than ASD females. In contrast to females, a statistically significant difference between the two male groups was observed for all tasks (according to non-parametric Kruskal–Wallis and *t*-test). ASD males shift significantly to lower values of the index of motor autonomy across all tasks, but visibly socio-motor tasks are deeply affected.

### 3.6 Motor controllability of an agent in a dyadic social interaction is inversely proportional to motor autonomy: leveraging socio-motor agency to protect the agent

In the Methods section, we defined what transfer entropy $T_{Y\to X}\left(k,l\right)$ between two processes X and Y is:


(6) $$T_{Y\to X}\left(k,l\right)=E\left[t_{Y\to X}\left(n+1,k,l\right)\right]$$

$$t_{Y\to X}\left(n+1,k,l\right)=i\left(y_{n}^{\left(l\right)};|x_{n+1}|x_{n}^{\left(k\right)}\right)$$

Equivalently, TE can be seen as the difference between the conditional entropy rate (which is equal to entropy rate for stationary processes) $h_{X}$ of process X and the generalized entropy rate $h_{X,Y}$ of X conditioning on the source Y (Prokopenko et al., 2013):


(7) $$T_{Y\to X}\left(k,l\right)=h_{X}-h_{X,Y}$$

With:


(8) $$h_{X}=-\sum p\left(x_{n+1},x_{n}^{\left(k\right)}\right)\log p\left(x_{n+1}|x_{n}^{k}\right)$$

(9) $$h_{X,Y}=-\sum p\left(x_{n+1},x_{n}^{\left(k\right)},y_{n}^{\left(l\right)}\right)\log p\left(x_{n+1}|,x_{n}|,y_{n}^{\left(l\right)}\right)$$

The generalized entropy rate measures the uncertainty in predicting the future values of X, given its history and the past values of Y. Transfer entropy is the reduction in uncertainty of predicting the future of X when we consider the process Y. If we call $h_{Y,X}$ uncertainty, then $h_{Y}$ is what we already defined as motor autonomy and $T_{X\to Y}$ is the transfer entropy.

We chose embedded history of length 20 for TE and for the entropy rate of our processes, we used a template (embedding) length equal to the average distance between two spikes to ensure that in the reconstructed space, the coordinates of a point in time include both zeros (“quiet moments”) and activity spikes and that the system does not bounce back and forth from a single coordinate of zero components. The embedding delay was chosen using average mutual information.

**Figure 7:**
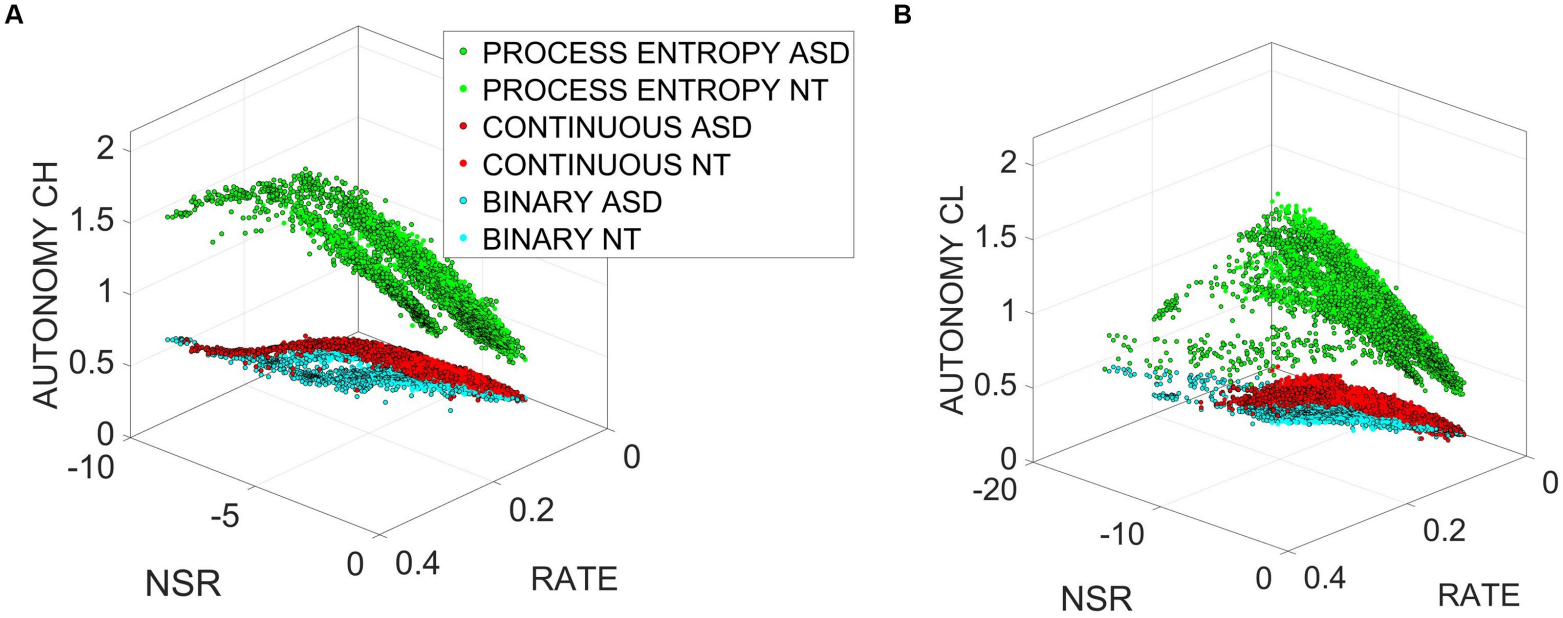
Non-i.i.d. process revealed by the relationship between autonomy, NSR, and spike rate for clinician **(A)** and child **(B)** for the Gamma and binary components of the MMS, relative to the process entropy.

If we plot the child or clinician autonomy with respect to the log(NSR) and the spike rate, we see in Figure 7 that the relationship between entropy rate, noise, and spike rate is rather complex. It also differs between NT and ASD; more data are needed to get a clear picture, but we can see that there is a small positive trend with respect to noise and spike rate. Nonetheless, this shows that the processes cannot be treated as an independent and identically distributed *i.i.d.* random process.

Now that we have established the speed/peak activity independence and the positive correlation between entropy rate and NSR or spike rate, we are ready to study how TE behaves in the shared space of the child–clinician dyad.

**Figure 8:**
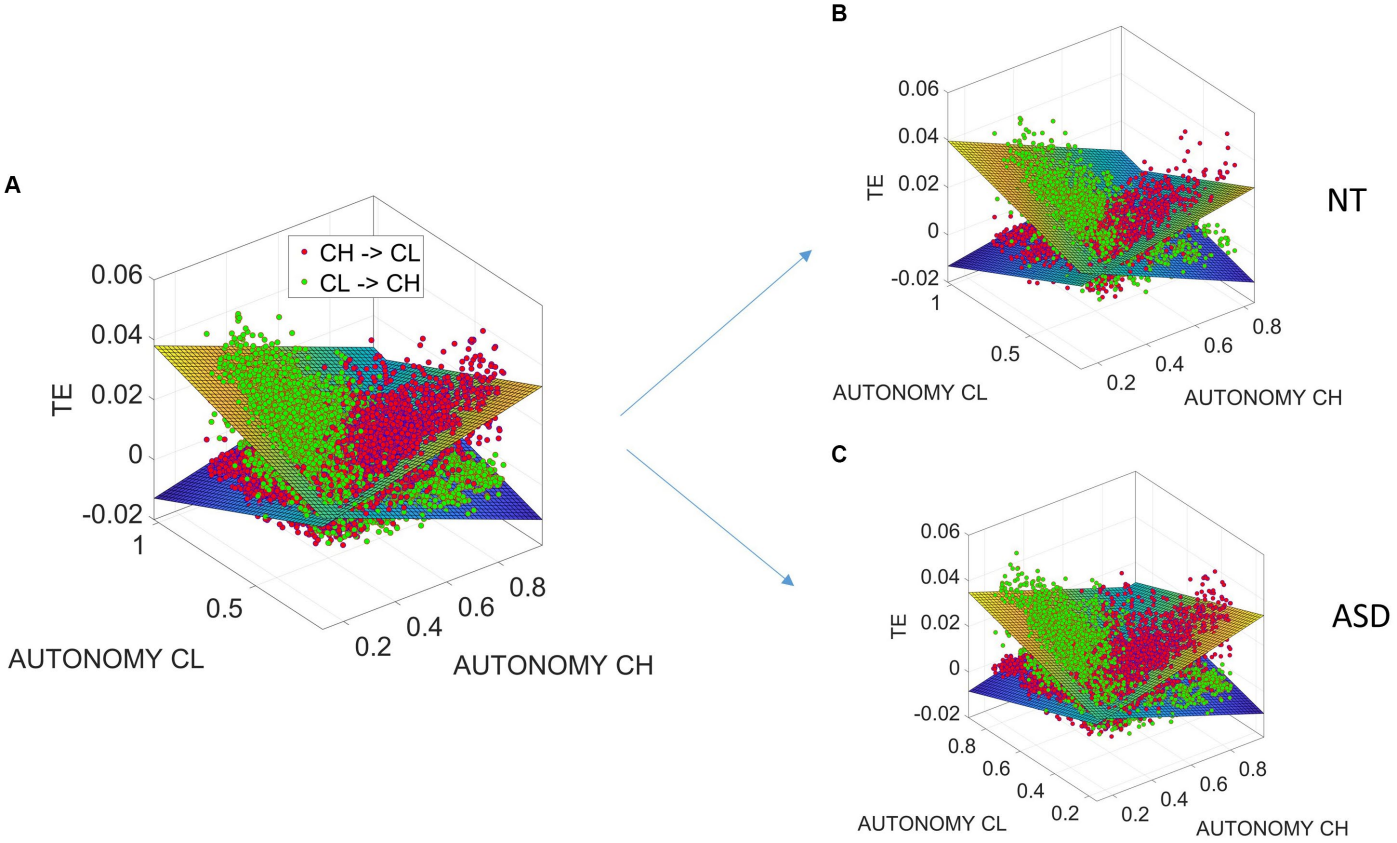
Linear relationships between transfer entropy and the entropy rates (autonomies) for child and clinician differentiating between NT and ASD participants. **(A)** Linear relationships between transfer entropy and the entropy rates (autonomies) for child and clinician. **(B)** Different linear relationships for NT and ASD participants.

We find that $TE_{CL\to CH}$ decreases when the child exhibits high motor autonomy and increases when the clinician has higher motor autonomy and vice versa for $TE_{CH\to CL}$ . In fact, this relationship is well characterized by linear relationships between transfer entropy and the entropy rates (autonomies), as the fitted linear surfaces indicate in Figure 8.

In this sense, we can safely conclude that by manipulating standardized human biorhythmic time series derived from human movements, either by increasing the NSR or by increasing peak activity, we can increase autonomy and reduce the controllability of human agents by other humans or by artificial agents, including those potentially created by AI. We return to this point in the concluding discussion and future work section of the article.

### 3.7 Validation of the digitization of the ADOS: automated, streamlined, and scalable screener of socio-motor agency

To make our basic scientific results actionable, we need to validate our digital data with clinical criteria, a paradigm that we have coined *clinically interpretable digital biomarkers*. In this model, the objective digital indexes that we used to define socio-motor agency as the motor autonomy-to-motor control ratio are examined in relation to the ADOS clinical scores that a trained (accredited) human rated during the session (while being naïve to the goals of the research). We employ a machine learning technique, support vector machine (SVM), to classify the digital data as a function of the clinical score. Then, we apply tools from signal detection theory, specifically the receiving operating characteristic curve, ROC, to assess the validity of our classifier.

Each of the 26 participants with the full ADOS session (digital and clinical) produces on average between 50 and 60 min of time series digital data from biosensors registering motion at 128 Hz. We used the left-hand wrist sensor in these analyses, as we showed that it is highly correlated with the right wrist, yet more variable, thus expanding our sampling space. As mentioned, upon exploration of several time windows to segment the data, sweeping across the time series and tasks while maximizing statistical power in each locally stationary segment, we arrived at 7.8-s windows as optimal.

The data were validated using the leave-one-person-out cross-validation (LOOCV) method. As features for our classifier, we used motor autonomy (entropy rate), motor control (NSR), Poisson binary micro-movement spike rate, transfer entropy, and the embedding delay of the data, which is the time scale at which deterministic properties arise and characterize the dynamical behavior of motion (for more information, see Methods). Two classifiers were used, one trained on female participants and the second one trained exclusively on male participants. When trying to digitally diagnose autism in one participant, we trained our classifier on the data from the remaining male or female participants and then tested how accurately the trained model predicted the participant class (NT vs. ASD). This method avoids overfitting and trains models that can diagnose autism in novel participants, thus automating the screening process.

**Figure 9:**
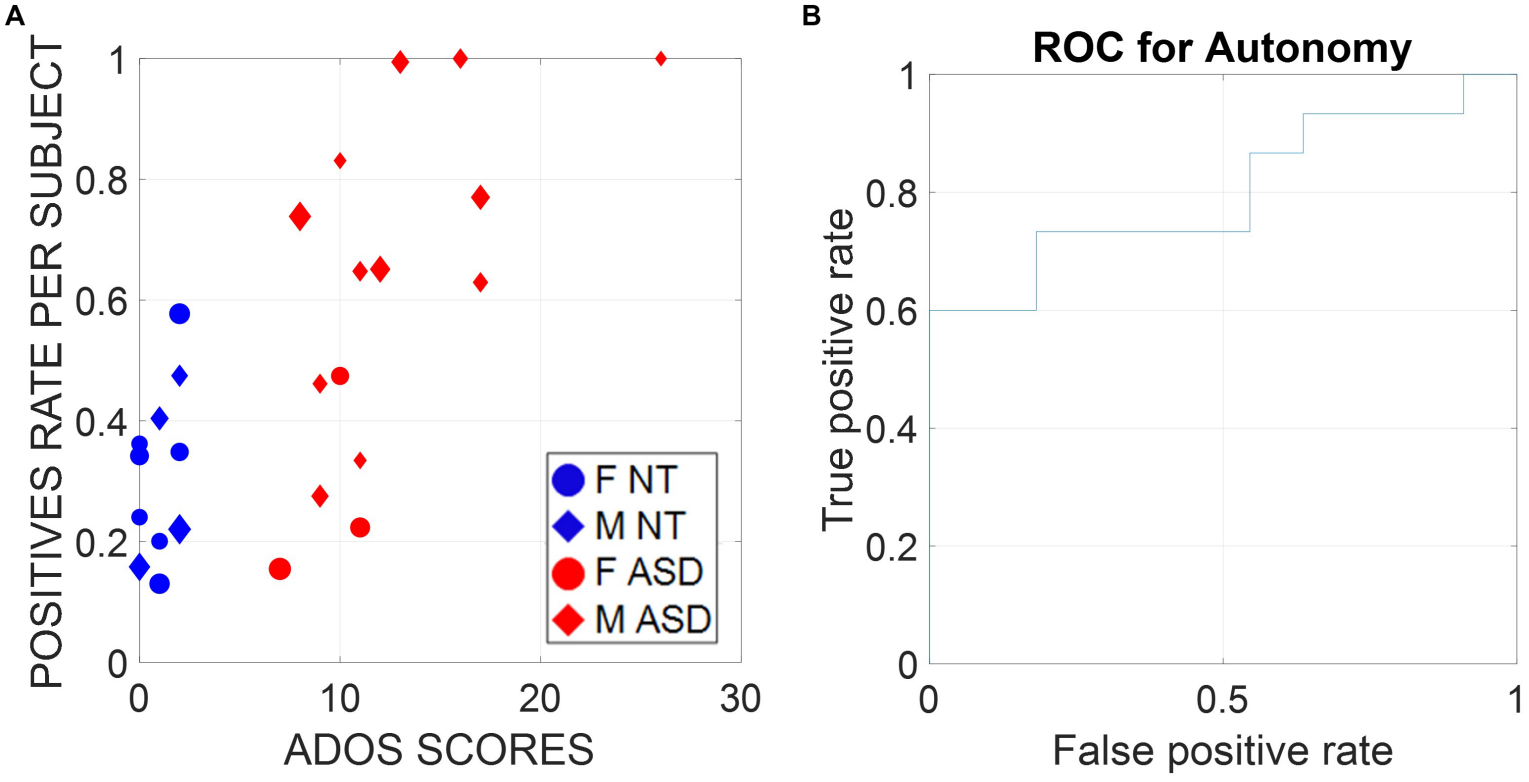
**(A)** Support vector machine (SVM) classifiers were trained on all subjects except one and tested on the remaining subjects of the same sex (leave-one-person-out cross-validation [LOOCV]). Therefore, each of the 26 subjects was digitally diagnosed with a classifier trained on a different dataset, which ensured zero overfitting and bias. Training and testing features were the entropy rate, the signal-to-noise ratio, transfer entropy, Poisson rate, and the embedding delay (the time scale at which a dynamical system behaves in the most deterministic way) calculated on normalized speed samples of ~7.8-s duration windows. Here, we report the percentage of time windows per subject that gave a positive diagnostic label and plot them versus the ADOS scores, as determined by the clinicians. **(B)** We use the positive rate scores as a metric used to diagnose ASD and report the receiver operating characteristic curve (ROC curve), which shows the true positive and false positive rates of the digital diagnostic tool we developed for different thresholds. The area under the curve (AUC) is 0.8, which indicates great performance.

Digitizing the ADOS in this way makes the diagnosis of autism more inclusive of females, historically underdiagnosed by a test that we objectively showed has biases toward males across all tasks (Torres et al., 2020b). A larger sample size and a longitudinal study are required to validate our model at scale. Yet, as shown in Figure 9A, there is no confusion of our biometrics about the clinician’s ADOS scores, which classify ASD males with 100% accuracy and perform remarkably well for ASD versus NT females. Indeed, Figure 9B confirms the validity of these biometrics for clinical use with an area under the ROC curve of 80%. Supplementary Figure S3 shows the set-up following standardized guidelines and materials from the official ADOS kit used in the research-grade version of the test.

## 4 Conclusion and future research

In this proof-of-concept work, we use the ADOS test as a backdrop to study social interactions between children and adult clinicians with the purpose of defining new ways to automate and speed up the autism screening process while leveraging the clinical validity of this test. To that end, we explored a new concept of socio-motor agency by defining a ratio of two indexes of motor autonomy and motor control. Motor autonomy was defined as the non-parametric entropy rate spanning from totally random to totally deterministic behavior of standardized micro-movement spike trains. These were derived from nuanced fluctuations in motion data that contain goal-directed segments of behavior interspersed with spontaneously occurring, more ambiguous, transient segments that are known to interconnect the goal-directed ones (Torres, 2011a, 2013). Motor control was defined with an eye for feedback-based predictability in terms of the NSR, empirically estimated from such spike trains as well. High regimes of NSR correspond to the memoryless exponential distribution regimes, denoting noisy feedback giving rise to high uncertainty (poor predictability and randomness) in the motor (reafferent) code.

We reasoned that these binarized sequences of spikes bear a motor code whereby the observer may or may not be able to predict the consequences of the observed actions by the agent and therefore be unable to control the observed agent. At high randomness, then, the observed agent affords more autonomy than at deterministic ranges. At deterministic ranges, with high regularity, the observer can predict and control the actions of the observed agent. At higher NSR, the agent has lower self-control. This is so because the kinesthetic reafferent feedback from the motions is noisy, and with such poor signal quality, it is difficult to predict a desired outcome and plan the action consequences to compensate for sensory transduction, transmission, and motor integration delays inherent in the person’s system. As predicting his/her/their motor action consequences can then be compromised by noise in the motor code, the child is more controlled by the clinician. The observer clinician can exert higher control over the observed agent. In this sense, the child’s socio-motor agency may also be compromised. This is the case whether the child/adult is autistic.

### 4.1 Key distinction between indexes of autonomy and control

It is important to distinguish between two levels of autonomy and control, defining the index of socio-motor agency. One is defining these components with respect to the self. In this case, we refer to the standalone analysis of the person’s time series biorhythmic data. The other one is in a social context, obtaining these indexes in relation to another agent who is interacting with the person.

In the case of self, self-autonomy is defined in relation to the systems’ autonomic, reflexive, and involuntary functions that attain a level of autonomous functionality and lend independence and self-reliance to the person’s nervous system. We defer this level of description and quantification for a different manuscript in this research topic (by Torres EB, *Gaining Insights into the Autistic Inner-experience Through a Personalized Digital Lens*). In the case that we characterize here within a social context involving another agent, autonomy is defined in relation to the amount of control that the external agent can exert over the person. In this sense, the use of the entropy rate as a proxy of the amount of autonomy that the person’s system has ranges from totally deterministic (the person’s rhythms are fully predictable and, as such, controllable by an external agent) to totally random (chaotic) and, as such, unpredictable and uncontrollable by an external agent. To further distinguish the index of autonomy from the proposed index of control, we verified experimentally that autonomy, as we define it, is directly related to predictive causality, as it is measured by the transfer entropy between the two biorhythmic time series—the person’s and the external agent’s causal influences on each other.

The case of the index of control is strictly defined at the individual (self) level. This biometric index is defined as the inverse of the empirically estimated noise-to-signal ratio (NSR) that we have examined across the human lifespan. Self-control undergoes an ontogenetic maturation process on a schedule (Torres et al., 2013a) well characterized by the shape and the scale parameters of the continuous Gamma family of probability distributions. Empirically, in human maturation, the ranges of these parameters span from the memoryless exponential in early ages (toddlers) to skewed distributions with heavy tails (school age) to the symmetric Gaussian (from college age onward). As the shape increases value with maturation and age, the NSR decreases. In old age and pathologies of the motor systems, the shape value declines back to exponential ranges and the NSR increases with different rates in disorders of the motor systems [e.g., Parkinson’s disease and neuronopathy due to deafferentation (Choi and Torres, 2014)]. As there is a scaling power law, linearly co-relating, on the log–log Gamma parameter plane these distribution parameters, we can see that knowing the shape (exponential to skewed to Gaussian) helps us accurately infer the scale, which in the Gamma case is the NSR. The shape systematically increases as the NSR systematically decreases. As such, we can define control in terms of the inverse of the NSR, i.e., the signal-to-noise ratio (SNR), and speak of predictability ranges spanning from memoryless random (no priors, just in the here and now) to Gaussian priors helping a predictive code.

To avoid any confusion between the control index defined by the NSR and the autonomy index, entropy rate (randomness), we underscore that ER is examined in relation to TE between the person’s and the external agent’s biorhythmic time series. We note that the NSR measures the variability of the person’s motions relative to the (empirically determined) baseline mean, given a situation or context. That is, this is a personalized measure of the person’s motions relative to the person’s baseline contextual variability. In contrast, entropy rate measures the empirically determined randomness of the person’s motions relative to an external agent, explicitly in the time domain. Although in the standalone (self) case of the micro-movement spikes, the NSR also informs or the levels of randomness/predictability of the biorhythmic code (Brincker and Torres, 2013), the two metrics, defining control and autonomy, are not generally equivalent concepts, as one can appreciate in Figure 5F.

Underlying both indexes and the ratio of motor autonomy to motor control are then discrete pockets of information making up a continuous stream of dyadic motor code, contributed by both social agents. Thus, we can infer the existence of an underlying shared alphabet in the motor code that manifests during dyadic social interactions of the type studied here. Agents with discrete motor signatures that appear more random are thus harder to control and behave more autonomously and independently than agents with systematically predictable motions sharing their codes.

### 4.2 Some notes on dyadic exchange and clinical applications

In addition to describing new biometrics of shared socio-motor agency in dyadic social interactions, our analyses showed ways to streamline the ADOS test, thus making it less taxing on the child and the clinician. A handful of tasks affording more socio-motor agency to the child can indeed uncover the social readiness potential of the child rather than biasing the diagnosis by the clinician toward a deficit model. Along those lines, using these newly defined indexes of dyadic motor autonomy and motor control, we demonstrated fundamental differences across the tasks for males and females, thus confirming that despite previously quantified differences in motor control separating males and females at the voluntary (Torres et al., 2013b) and involuntary (Torres et al., 2016a, 2017) levels, the ADOS remains biased toward males.

The proposed digital indexes of shared socio-motor agency, used within the context of an unbiased ML classifier, could detect the differences between males and females for both the NT and ASD randomly chosen participants. While the proposed indexes speak of motor physiology underlying socio-motor agency during social interactions, psychological constructs of social interactions and communication defining the ADOS test were also leveraged by the presented ML methods. This digitized, automated version of the test resembles the type of scenario that a clinician faces at the clinic on any given day. Namely, a random arrival of a case that the clinician may see for the first time. In that sense, the leave-one-person-out classifier provides robust digital screening of autism and may be a way, in future research, to scale our pilot study to encompass larger numbers of NT and ASD participants across ages and sexes and do so longitudinally as well. This type of approach combining traditional psychological and newly emerging physiological motor criteria of autism could help us close the gap between disparate literatures of autism spanning several decades (Whyatt and Torres, 2018).

Future longitudinal studies of autism with an eye for the evolution of the neuromotor code and its impact on social perception and cognition will require the type of normalization that we introduced earlier with the MMS (Torres et al., 2013a) and further used here in the dyadic context, namely, scaling out allometric effects due to anatomical differences across participants (see also Torres et al., 2016a,b, 2020a; Caballero et al., 2020). This step is crucial in any study that involves biorhythmic motions, whereby kinematic analyses will be impacted by such anatomical differences. This is so because kinematic parameters such as speed, acceleration, distance, etc. are impacted by the limb sizes and masses in ways that confound results and interpretation of such studies (Torres et al., 2018). It will be particularly important to consider these caveats present in all current studies of kinematics that do not account for allometric differences during the very early neurodevelopment when the rate of bodily growth is highly non-linear and accelerated (Torres et al., 2016b). These rates of changes in anatomical growth produce different ranges of values in such kinematic parameters and impact the empirical distributions of the values associated with natural behaviors such as those examined here (Torres et al., 2016b, 2018).

### 4.3 Implications of socio-motor agency metrics for AI and privacy protection

The theoretical considerations at the intersection of stochastic analyses and information-theoretic approaches with non-linear dynamics offer the MMS and Gamma analyses as a viable way to obtain the personalized signatures of socio-motor autonomy and socio-motor control and tweak the NSR to mask the spike trains derived from the person’s physiological biorhythmic activity. This ability to separate the binary spike rate code from the Gamma process, denoting levels of randomness versus predictability, offers the possibility of creating a device that alerts the persons involved in the dyadic exchange to balance their autonomy and control and to attain socio-motor agency. By enhancing autonomy and avoiding excessive external control by the other agent, be that agent another human or an AI-driven one, the person can be protected from excess control. This approach will be critical to revamp autism therapies with an emphasis to respect the child’s autonomy and support the bottom-up development of autonomous motor control. The maturation of bottom-up autonomous motor control (building blocks of the person’s psychological sense of autonomy) is a necessary pre-requisite for the further neurodevelopment of top-down cognitive control. Without considering and balancing the orderly maturation rates of these two building blocks of socio-motor behavior, therapies in autism are bound to stunt the natural development of socio-motor agency and likely cause trauma to the nascent nervous system.

We propose that this methodology can also be used to protect our privacy more generally from surveillance systems, as ultimately these systems rely on biometric data, which we can now, using the present personalized methods, manipulate to hide our fingerprint-like bio-motor signatures from an external agent trying to control us. This solution to the controllability issue can then be extended from individuals to dyads, from dyads to social groups, and from social groups to society. In this sense, socio-motor agency can serve as a foundation for societal agency, now quantifiable using the methods that we offer in this study.

### 4.4 Limitations

Our study has a modest number of participants. Although it provides proof-of-concept that we can digitize natural dyadic interactions, it will be necessary to reproduce our study with a larger N. To that end, we provide our code and data samples for independent reproducibility by other groups with access to more participants.

In summary, we found that variability in the dyadic index of motor autonomy is more pronounced in ASD than in NTs, across a broad range of ages from 4 to 15 years old. Furthermore, we found that the dyadic NSR, indicative of socio-motor control, increases with age. This result is consistent with prior work on individuals across ages and sex (Torres et al., 2013a,b). In contrast, both ASD and NT showed increases in the motor autonomy index with age, an indicator that regardless of the human condition, whether developing along a neurotypical trajectory or along the trajectory toward autism spectrum disorders, respecting the child’s autonomy will be necessarily our best ally when designing future treatments that unveil the child’s social readiness potential. We would not have known this had we treated the ADOS as the criterion test that it is (i.e., exclusively based on children with neurodevelopmental differences), rather than treating it as a normative test (i.e., including NT controls as well, to define normative ranges and quantify similarities and departures from it).

We have uncovered new indexes of shared, dyadic motor autonomy and motor control, objectively defined socio-motor agency, and provided new means to automate its digital screening with already routinely used clinical tools. This study offers novel ways to scale our clinical science and make it actionable, diverse, and inclusive at more than one level.

## Data Availability

The datasets presented in this study can be found in online repositories and will be available upon request from the authors. The names of the repository/repositories and accession number(s) can be found at: https://zenodo.org/records/10032169.

## References

[ref1] Accardo P. J. Barrow W. (2015). Toe walking in autism: further observations. J. Child Neurol. 30, 606–609. doi: 10.1177/0883073814521298 24563477

[ref2] Allen G. Muller R. A. Courchesne E. (2004). Cerebellar function in autism: functional magnetic resonance image activation during a simple motor task. Biol. Psychiatry 56, 269–278. doi: 10.1016/j.biopsych.2004.06.005, PMID: 15312815

[ref3] Bermperidis T. Rai R. Ryu J. Zanotto D. Agrawal S. K. Lalwani A. K.. (2021). Optimal time lags from causal prediction model help stratify and forecast nervous system pathology. Sci. Rep. 11:20904. doi: 10.1038/s41598-021-00156-2, PMID: 34686679 PMC8536772

[ref4] Bokadia H. Rai R. Torres E. B. (2020). Digitized autism observation diagnostic schedule: social interactions beyond the limits of the naked eye. J. Pers. Med. 10, 1–25. doi: 10.3390/jpm10040159, PMID: 33050080 PMC7711822

[ref5] Brincker M. Torres E. B. (2013). Noise from the periphery in autism. Front. Integr. Neurosci. 7:34. 23898242 10.3389/fnint.2013.00034 PMC3721435

[ref6] Brincker M. Torres E. B. (2018). “Chapter 1-why study movement variability in Autism” in Autism: The movement sensing perspective. eds. Torres E. B. Whyatt C. (Boca Raton: CRC Press/Taylor & Francis Group). pp. xviii, 386 pages.

[ref7] Caballero C. Mistry S. Torres E. B. (2020). Age-dependent statistical changes of involuntary head motion signatures across autism and controls of the ABIDE repository. Front. Integr. Neurosci. 14, 1–14. doi: 10.3389/fnint.2020.00023 32625069 PMC7311771

[ref8] Choi K. Torres E. B. (2014). Intentional signal in prefrontal cortex generalizes across different sensory modalities. J. Neurophysiol. 112, 61–80. doi: 10.1152/jn.00505.2013, PMID: 24259543

[ref9] Chukoskie L. Townsend J. Westerfield M. (2013). Motor skill in autism spectrum disorders: a subcortical view. Int. Rev. Neurobiol. 113, 207–249. doi: 10.1016/B978-0-12-418700-9.00007-1 24290387

[ref10] Cornelio P. Haggard P. Hornbaek K. Georgiou O. Bergstrom J. Subramanian S.. (2022). The sense of agency in emerging technologies for human-computer integration: a review. Front. Neurosci. 16:949138. doi: 10.3389/fnins.2022.949138, PMID: 36172040 PMC9511170

[ref11] Delgado-Bonal A. Marshak A. (2019). Approximate entropy and sample entropy: a comprehensive tutorial. Entropy (Basel) 21, 1–21. doi: 10.3390/e21060541 PMC7515030 33267255

[ref12] D'Mello A. M. Frosch I. R. Li C. E. Cardinaux A. L. Gabrieli J. D. E. (2022). Exclusion of females in autism research: empirical evidence for a "leaky" recruitment-to-research pipeline. Autism Res. 15, 1929–1940. doi: 10.1002/aur.2795, PMID: 36054081 PMC9804357

[ref13] D'Mello A. M. Stoodley C. J. (2015). Cerebro-cerebellar circuits in autism spectrum disorder. Front. Neurosci. 9:408. doi: 10.3389/fnins.2015.00408 26594140 PMC4633503

[ref14] Elsayed M. Torres E. B. (2023). Exploring cardiac responses of pain and distress, topics in autonomic nervous system. London: Intech Open.

[ref15] Fournier K. A. Hass C. J. Naik S. K. Lodha N. Cauraugh J. H. (2010). Motor coordination in autism spectrum disorders: a synthesis and meta-analysis. J. Autism Dev. Disord. 40, 1227–1240. doi: 10.1007/s10803-010-0981-3, PMID: 20195737

[ref16] Frosch I. R. Mittal V. A. D'Mello A. M. (2022). Cerebellar contributions to social cognition in ASD: a predictive processing framework. Front. Integr. Neurosci. 16:810425. doi: 10.3389/fnint.2022.810425, PMID: 35153691 PMC8832100

[ref17] Gotham K. Risi S. Pickles A. Lord C. (2007). The autism diagnostic observation schedule: revised algorithms for improved diagnostic validity. J. Autism Dev. Disord. 37, 613–627. doi: 10.1007/s10803-006-0280-1, PMID: 17180459

[ref18] Hallett M. Lebiedowska M. K. Thomas S. L. Stanhope S. J. Denckla M. B. Rumsey J. (1993). Locomotion of autistic adults. Arch. Neurol. 50, 1304–1308. doi: 10.1001/archneur.1993.00540120019007 8257307

[ref19] Hollander M. Pena E. A. (2004). Nonparametric methods in reliability. Stat. Sci. 19, 644–651. doi: 10.1214/088342304000000521, PMID: 16710444 PMC1463894

[ref20] Lizier J. T. (2014). JIDT: an information-theoretic toolkit for studying the dynamics of complex systems. Front. Robot. AI 1, 1–20. doi: 10.3389/frobt.2014.00011

[ref21] Lleonart J. Salat J. Torres G. J. (2000). Removing allometric effects of body size in morphological analysis. J. Theor. Biol. 205, 85–93. doi: 10.1006/jtbi.2000.2043, PMID: 10860702

[ref22] Loomes R. Hull L. Mandy W. P. L. (2017). What is the male-to-female ratio in autism Spectrum disorder? A systematic review and Meta-analysis. J. Am. Acad. Child Adolesc. Psychiatry 56, 466–474. doi: 10.1016/j.jaac.2017.03.013, PMID: 28545751

[ref23] Lord C. Risi S. Lambrecht L. Cook E. H. Jr. Leventhal B. L. DiLavore P. C.. (2000). The autism diagnostic observation schedule-generic: a standard measure of social and communication deficits associated with the spectrum of autism. J. Autism Dev. Disord. 30, 205–223. doi: 10.1023/A:1005592401947 11055457

[ref24] Lundstrom S. Marland C. Kuja-Halkola R. Anckarsater H. Lichtenstein P. Gillberg C.. (2019). Assessing autism in females: the importance of a sex-specific comparison. Psychiatry Res. 282:112566. doi: 10.1016/j.psychres.2019.112566, PMID: 31558402

[ref25] Mohamed Thangal S. N. Donelan J. M. (2020). Scaling of inertial delays in terrestrial mammals. PLoS One 15:e0217188. doi: 10.1371/journal.pone.0217188, PMID: 32017765 PMC6999919

[ref26] Nayate A. Bradshaw J. L. Rinehart N. J. (2005). Autism and Asperger's disorder: are they movement disorders involving the cerebellum and/or basal ganglia? Brain Res. Bull. 67, 327–334. doi: 10.1016/j.brainresbull.2005.07.011 16182941

[ref27] Prokopenko M. Lizier J. T. Price D. C. (2013). On thermodynamic interpretation of transfer entropy. Entropy 15, 524–543. doi: 10.3390/e15020524

[ref28] Sivalingam A. M. Pandian A. (2024). Cerebellar roles in motor and social functions and implications for ASD. Cerebellum. 30, 606–609. doi: 10.1007/s12311-024-01720-y 39017808

[ref29] Somoza E. Mossman D. (1991). ROC curves and the binormal assumption. J. Neuropsychiatry Clin. Neurosci. 3, 436–439. doi: 10.1176/jnp.3.4.436 1821267

[ref30] Torres E. B. (2011a). Two classes of movements in motor control. Exp. Brain Res. 215, 269–283. doi: 10.1007/s00221-011-2892-8 22038712

[ref31] Torres E. B. (2011b). Atypical signatures of motor variability found in an individual with ASD. Neurocase 19, 150–165. doi: 10.1080/13554794.2011.654224 22587379

[ref32] Torres E. B. (2013). Signatures of movement variability anticipate hand speed according to levels of intent. Behav. Brain Funct. 9:10. doi: 10.1186/1744-9081-9-10, PMID: 23497360 PMC3635904

[ref33] Torres E. B. Brincker M. Isenhower R. W. Yanovich P. Stigler K. A. Nurnberger J. I.. (2013a). Autism: the micro-movement perspective. Front. Integr. Neurosci. 7:32. doi: 10.3389/fnint.2013.00032 23898241 PMC3721360

[ref34] Torres E. B. Caballero C. Mistry S. (2020a). Aging with autism departs greatly from typical aging. Sensors (Basel) 20, 1–21. doi: 10.3390/s20020572, PMID: 31968701 PMC7014496

[ref35] Torres E. B. Denisova K. (2016). Motor noise is rich signal in autism research and pharmacological treatments. Sci. Rep. 6:37422. doi: 10.1038/srep37422, PMID: 27869148 PMC5116649

[ref36] Torres E. B. Donnellan A. M. (2015). Editorial for research topic "autism: the movement perspective". Front. Integr. Neurosci. 9:12. doi: 10.3389/fnint.2015.00012 25852501 PMC4360560

[ref37] Torres E. B. Isenhower R. W. Nguyen J. Whyatt C. Nurnberger J. I. Jose J. V.. (2016a). Toward precision psychiatry: statistical platform for the personalized characterization of natural behaviors. Front. Neurol. 7:8. doi: 10.3389/fneur.2016.00008 26869988 PMC4735831

[ref38] Torres E. B. Isenhower R. W. Yanovich P. Rehrig G. Stigler K. Nurnberger J.. (2013b). Strategies to develop putative biomarkers to characterize the female phenotype with autism spectrum disorders. J. Neurophysiol. 110, 1646–1662. doi: 10.1152/jn.00059.2013, PMID: 23864377

[ref39] Torres E. B. Mistry S. Caballero C. Whyatt C. P. (2017). Stochastic signatures of involuntary head Micro-movements can be used to classify females of ABIDE into different subtypes of neurodevelopmental disorders. Front. Integr. Neurosci. 11:10. doi: 10.3389/fnint.2017.00010, PMID: 28638324 PMC5461345

[ref40] Torres E. B. Rai R. Mistry S. Gupta B. (2020b). Hidden aspects of the research ADOS are bound to affect autism science. Neural Comput. 32, 515–561. doi: 10.1162/neco_a_01263, PMID: 31951797

[ref41] Torres E. B. Smith B. Mistry S. Brincker M. Whyatt C. (2016b). Neonatal diagnostics: toward dynamic growth charts of Neuromotor control. Front. Pediatr. 4:121. doi: 10.3389/fped.2016.00121, PMID: 27933283 PMC5120129

[ref42] Torres E. B. Varkey H. Vero J. London E. Phan H. Kittler P.. (2023). Sensing echoes: temporal misalignment in auditory brainstem responses as the earliest marker of neurodevelopmental derailment. PNAS Nexus 2:pgac315. doi: 10.1093/pnasnexus/pgac315, PMID: 36798622 PMC9927073

[ref43] Torres E. B. Vero J. Rai R. (2018). Statistical platform for individualized behavioral analyses using biophysical Micro-movement spikes. Sensors (Basel) 18. doi: 10.3390/s18041025, PMID: 29596342 PMC5948575

[ref44] Torres E. B. Whyatt C. (2018). Autism: The movement sensing perspective. Boca Raton: CRC Press/Taylor & Francis Group. 1–30.

[ref45] Torres E. B. Yanovich P. Metaxas D. N. (2013c). Give spontaneity and self-discovery a chance in ASD: spontaneous peripheral limb variability as a proxy to evoke centrally driven intentional acts. Front. Integr. Neurosci. 7:46. doi: 10.3389/fnint.2013.00046 23898243 PMC3721359

[ref46] Vilensky J. A. Damasio A. R. Maurer R. G. (1981). Gait disturbances in patients with autistic behavior: a preliminary study. Arch. Neurol. 38, 646–649. doi: 10.1001/archneur.1981.00510100074013, PMID: 7295109

[ref47] Weiss C. Tsakiris M. Haggard P. Schutz-Bosbach S. (2014). Agency in the sensorimotor system and its relation to explicit action awareness. Neuropsychologia 52, 82–92. doi: 10.1016/j.neuropsychologia.2013.09.034, PMID: 24096174

[ref48] Whyatt C. P. Torres E. B. (2018). Autism research: an objective quantitative review of Progress and focus between 1994 and 2015. Front. Psychol. 9:1526. doi: 10.3389/fpsyg.2018.01526, PMID: 30190695 PMC6116169

[ref49] Wu X. Dickin D. C. Bassette L. Ashton C. Wang H. (2024). Clinical gait analysis in older children with autism spectrum disorder. Sports Med. Health Sci. 6, 154–158. doi: 10.1016/j.smhs.2023.10.007, PMID: 38708319 PMC11067783

[ref50] Wu D. Jose J. V. Nurnberger J. I. Torres E. B. (2018). A biomarker characterizing neurodevelopment with applications in autism. Sci. Rep. 8:614. doi: 10.1038/s41598-017-18902-w, PMID: 29330487 PMC5766517

[ref51] Ziv I. Avni I. Dinstein I. Meiri G. Bonneh Y. S. (2024). Oculomotor randomness is higher in autistic children and increases with the severity of symptoms. Autism Res. 17, 249–265. doi: 10.1002/aur.3083, PMID: 38189581

