# Supplementary material for: Digital screener of socio-motor agency balancing motor autonomy and motor control
Source: Front Hum Neurosci. 2024 Oct 1;18:1442799. doi: 10.3389/fnhum.2024.1442799 (PMC11473353; doi:10.3389/fnhum.2024.1442799)
Supplement: Supplementary file 1 [file Data_Sheet_1.PDF]

Supplementary Material

1 Supplementary Concepts and Equations from Section 2 of the Main Paper

The following equations follow from Section 2 of the Main Paper and will be numbered accordingly.

1.1 Binary Trains of MMS

If we transform the MMS data so that the presence of a peak corresponds to the binary "1" and an absence of a peak corresponds to the binary "0", we can represent normalized speed (or acceleration) as a stochastic binary sequence. An underlying mechanism stochastically generates bursts of activity, and this is equivalent to randomly generating 0s and 1s from an underlying binary alphabet as in Equation 2.6.

$$B_t = 1, \text{ if } NormPeak_t > threshold \qquad \text{Equation (2.6)}$$

Here the threshold of 0 value represents the empirically estimated Gamma mean, particular to the person within the context. Let's assume that B_t is a random sample drawn from an underlying probability distribution at time t.

$$B_t = 1 \text{ with probability } p_t, 0 \text{ with probability } 1 - p_t \qquad \text{Equation (2.7)}$$

Entropy H in Equation 2.8 is an information theoretic measure that quantifies the amount of information in a random variable that follows a probability distribution P_x [39] and is equal to:

$$H = -\sum_x p_x \ log_a \, p_x \qquad \text{Equation (2.8)}$$

In the case of the binary process, the amount of information of the random variable of an activity outburst (MMS) is given in Equation 2.9:

$$H_t = -p_t \, log_a(p_t) - (1 - p_t) \, log_a(1 - p_t) \qquad \text{Equation (2.9)}$$

Which takes the maximum value of 1 when $p_t = 0.5$ and the minimum value of 0, when $p_t = 0$ or 1. Intuitively, entropy measures either the uncertainty regarding the outcome of a random realization of the random variable before that variable is measured or equivalently, the amount of information we get when we observe the variable. If we know for example, that with a 100 % chance $B_t = 1$, the entropy is zero as we have no uncertainty about the outcome of the measurement, and no valuable information is provided to us. However, if with a 50 % chance $B_t = 1$, the entropy is at its maximum because we are totally uncertain whether the outcome will be 0 or 1 and observing the outcome gives us maximal information, specifically, 1 bit of information in the case of a base 2 logarithm ($a = 2$).

1.2 Measuring Randomness *vs.* Predictability Using Entropy Rate

The definition of entropy can be generalized for the case of multiple random variables $X_1, X_2, \dots, X_N$, as in equation 2.10, by considering the joint probability distribution $P_{X_1,X_2,\dots,X_N}$:

$$H(X_1, X_2, \dots, X_N) = -\sum_{X_1,X_2,\dots X_N,} P_{X_1,X_2,\dots,X_N} \log_a(P_{X_1,X_2,\dots,X_N}) \qquad \text{Equation (2.10)}$$

In the case of a stationary stochastic process X (*i.e.*, statistical properties preserved over time) which takes values from a discrete alphabet K (in the case of the binary MMS), we can define the entropy rate of the process as in Equation 2.11:

$$H(X) = \frac{1}{T} \lim_{T\to\infty} H(X_1, X_2, \dots, X_T) \qquad \text{Equation (2.11)}$$

This quantity measures how much the process changes over time, *i.e.,* the information that is carried in a new value. It measures the degree of randomness (unpredictability) of the underlying dynamical system [39; 40; 41; 42].

1.3 Randomness for Dynamical Systems

The concept of entropy rate is not limited to random processes, but it can also be defined in the case of deterministic dynamical systems. Let x_t be a continuous univariate times series. Then we can construct a state-space representation of the process as in Equation 2.12 if we choose an appropriate dimension d of the presumed underlying dynamical system and an embedding delay τ [43; 44].

$$X_t = \begin{pmatrix} x_1 \\ x_2 \\ . \\ . \\ . \\ x_d \end{pmatrix}_t = \begin{pmatrix} x(t) \\ x(t+\tau) \\ . \\ . \\ . \\ x(t+(d-1)\tau) \end{pmatrix} \quad \text{Equation (2.12)}$$

The existence and calculations of the embedding dimension and delay are ensured by Taken's embedding theorem [45]. For more information on dynamical systems theory see *e.g.*, [46; 47]. Essentially, any univariate time series can be viewed as being sampled from a high dimensional dynamical system [48]. The dynamical system follows a trajectory in the d-dimensional space defined by the d degrees of freedom. All possible states of the dynamical system define the phase space of the system.

If we partition the phase space across F dimensions, with $F \leq d$, we have an F-dimensional grid of cells of volume r^F. Then, we can measure the state of the system at constant time intervals equal to the embdedding delay τ. We can define the joint probability $p(i_1, i_2, \dots, i_d)$ that X_τ is in cell i_1, $X_{2\tau}$ is in cell i_2,…, $X_{d\tau}$ is in cell i_d. The degree of "randomness" of the deterministic system can then be calculated using the Kolmogorov-Sinai (KS) entropy[49] using Equation 2.13:

$$KS = -\lim_{\tau\to 0} \lim_{r\to 0} \lim_{d\to\infty} \frac{1}{d\tau} \sum_{i_1,i_2,\dots,i_d} p(i_1, i_2, \dots, i_d) \log_a \; p(i_1, i_2, \dots, i_d) \quad \text{Equation (2.13)}$$

The KS entropy is almost always equal to the entropy rate of the original signal x_t and characterizes the degree of randomness of the system (and subsequently the sampled one-dimensional signal). For completely deterministic systems it is equal to zero and it is infinite for random systems.

In practice, the entropy rate is approximated using what is known as the correlation integral [50] in Equation 2.14:

$$C_d(r) = \lim_{N\to\infty} \frac{1}{N^2} [\# \, (n,m), \big(\textstyle\sum_{i=1}^{d} |X_{n+i} - X_{m+i}\,|^2\big)^{\frac{1}{2}} \leq \; r\,] \quad \text{Equation (2.14)}$$

i.e., the (#) number of pairs of trajectory points that are close to each within a tolerance threshold r and measures the regularity (frequency) of patterns like a given template of specific length.

It can be shown that:

$$\lim_{d\to \infty, r\to 0} \frac{1}{\tau} log_a \frac{C_d(r)}{C_{d+1}(r)} \sim K_2$$ Equation (2.15)

Where K_2 is the Renyi entropy of order 2. The Renyi entropy K_a in Equation 2.16 is a generalized form of the usual Shannon entropy and is defined as:

$$K_a = \frac{1}{1-a} log_a(\sum_x p_x^a)$$ Equation (2.16)

We leverage these tools to calculate the entropy rate in the case of a discrete time series $u(n)$. Consider two different blocks of length m sampled from the time series:

$$x(i) = \{u(i), u(i+1) \ldots, u(i+m-1)\}$$

$$x(j) = \{u(j), u(j+1), \ldots u(j+m-1)\}$$

And define the distance in Equation 2.17:

$$d[x(i), x(j)] = \max_{k=1,2,\ldots m} (|u(i+k-1) - u(j+k-1)|)$$ Equation (2.17)

i.e., the maximum distance between the two vectors (Chebyshev distance). Then, we can define a quantity in Equation 2.18 like the correlation integral, for a template of length m at $x(i)$ within a tolerance threshold r:

$$C_i^m = \frac{\#\ j \le N-m+1, d[x(i), x(j)] \le r}{N-m+1}$$ Equation (2.18)

Then, the entropy rate can be estimated as in Equation 2.19:

$$ER = \lim_{r\to 0} \lim_{m\to \infty} \lim_{N\to \infty} [\varphi^m(r) - \varphi^{m-1}(r)]$$ Equation (2.19)

Where as in [51] Equation 2.20 gives:

$$\varphi^m(r) = \frac{1}{N-m+1} \sum_{i=1}^{N-m+1} log_a\, C_i^m(r) \qquad \text{Equation (2.20)}$$

Since $C_i^m(r)$ is essentially the probability that any sequence of length m is very close to the template sequence at time i, and $C_i^{m-1}(r)$ the probability that the same holds true for sequences of length $m-1$, then $\frac{C_i^m(r)}{C_i^{m-1}(r)}$ is the conditional probability that any sequence of length m is very close to the template of length m at time i given that the same holds true for $m-1$. Then $log_a\left(\frac{C_i^m(r)}{C_i^{m-1}(r)}\right) = log_a\left(C_i^m(r)\right) - log_a(C_i^{m-1}(r))$ the logarithm of this conditional probability. It is easy to see that $\varphi^m(r) - \varphi^{m-1}(r)$ is the average over i of the logarithm of this conditional probability [49].

However, due to finite sample sizes and stochasticity in time series analysis, the entropy rate can be estimated by what is known as Approximate Entropy (ApEn) [46] and is given by Equation 2.21:

$$ApEn(m, r, N)(u) = \varphi^m - \varphi^{m-1} \qquad \text{Equation (2.21)}$$

Where N is the length of the time series $u(n)$, m is the choice of the length template and r is the threshold tolerance choice. Approximate entropy measures the logarithmic frequency with which segments of length m that are very close together (according to the threshold), stay together through time.

An approximate formula for ApEn, which we implemented in our study is given by Equation 2.22:

$$ApEn(m, r, N) \cong \frac{1}{N-m} \sum_{i=1}^{N-m} log_a \frac{\sum_{j=1}^{N-m}[\#\, j, d[|x_{m+1}(j) - x_{m+1}\,(i)|] < r]}{\sum_{j=1}^{N-m}[\#\, j, d[|x_m(j) - x_m\,(i)|] < r]} \qquad \text{Equation (2.22)}$$

1.4 Entropy Rate estimation for a binary MMS speed sequence

Generally, in the case of discrete alphabet sequences with k symbols, $0 \leq ApEn \leq log_a\, k$

Where $ApEn = 0$ for deterministic time series and $ApEn = log_a\, k$ for random series.

In our case (binary MM series), $k = 2$ and $0 \leq ApEn_{MM} \leq log_a 2$

For $a = e$ (natural logarithm choice), the maximum value is $ln(2) = 0.69$, which is the base we use in this study [49].

A good choice of m is equal to the embedding dimension, which can be estimated using the False Nearest Neighbor (FNN) algorithm[52]. Usually, m is of low dimension, in our case the dimension of the data was estimated to be 2. The threshold r is usually set between 0.1 to 0.25 standard deviations of the time series [49].

1.5 Quantifying Information Flow Between Binarized MMS with Local Transfer Entropy

Local Shannon Entropy is defined in Equation 2.23 as the negative logarithm of the probability of an outcome x of a random variable [53]:

$$h(x) = -\,log_2\, p(x) \qquad \text{Equation (2.23)}$$

where low probability outcomes carry more information than high probability outcomes. Entropy as defined in Equation 2.24 can then be expressed as the average value of all such outcomes:

$$H(X) = E[h(x)] = -\sum_x p(x)\, log_2\, p(x) \qquad \text{Equation (2.24)}$$

Where $E[.]$ is the expectation (average) operator. An estimator based on samples x_n is given by Equation 2.25:

$$H(x) \cong \frac{1}{N} \sum_{n=1}^{N} h(x_n) \qquad \text{Equation (2.25)}$$

The Local Mutual Information $i(x; y)$ and Mutual Information (MI) $I(X; Y)$ are respectively defined in Equations 2.26 and 2.27, [54]:

$$i(x;y) = log_2 \frac{p(x|y)}{p(x)} = h(x) - h(x|y) \quad \text{Equation (2.26)}$$

$$I(X;Y) = E[i(x;y)] \quad \text{Equation (2.27)}$$

Equation 2.27 quantifies the information that we gain when observing X after we have already observed another variable Y.

Similarly, the Local Conditional Mutual Information and Conditional Mutual Information are given by Equations 2.28 and 2.29 respectively, [54]:

$$i(x;y|z) = log_2 \frac{p(x|y,z)}{p(x|z)} = h(x|z) - h(x|y,z) \quad \text{Equation (2.28)}$$

$$I(X;Y|Z) = E[i(x;y|z)] \quad \text{Equation (2.29)}$$

It quantifies the information that we gain when we observe X after considering both Y and Z versus considering only Z.

Finally, local transfer entropy quantifies the flow of information from Y to X and is defined in Equation 2.30, [55; 56]:

$$t_{Y\to X}(n+1,k,l,u) = i(\boldsymbol{y}_{n+1-u}^{(l)}; x_{n+1}|\boldsymbol{x}_n^{(k)}) \quad \text{Equation (2.30)}$$

Where l and k denote the length of the vectors $\boldsymbol{y}_{n+1-u}^{(l)} = \{y_{n+1-u-l+1}, \dots, y_{n+1-u-1}, y_{n+1-u}\}$ (storing past information of the process Y with a memory of l samples up to point $n+1-u$) and $\boldsymbol{x}_n^{(k)} = \{x_{n-k+1}, \dots, x_{n-1}, x_n\}$.

The integer u denotes the source-destination lag, *i.e.*, the causal time delay between Y and X that we are interested in when we want to calculate the transfer entropy from Y to X. For $u = 1$, a typical choice of source-destination lag is given by Equation 2.31:

$$t_{Y\to X}(n+1,k,l) = i(\boldsymbol{y}_n^{(l)}; x_{n+1}|\boldsymbol{x}_n^{(k)}) \quad \text{Equation (2.31)}$$

The local transfer entropy is the mutual information between Y and the future state of X, u samples ahead, conditioned on the history of X. In other words, it measures the information gained that we get about the future state of X when considering both its own past and the past states of Y versus considering only its past state. Transfer entropy is the expected information gain, averaging over all states given by Equation 2.32, [53; 55]:

$$T_{Y\to X}(k,l) = E[t_{Y\to X}(n+1,k,l)] \qquad \text{Equation (2.32)}$$

1.6 Quantifying Autonomy of an Agent from the Perspective of the Observer

For a child/clinician dyad, we obtain the normalized MMS derived from the fluctuations in angular speed from the right- and left-wrist sensors throughout the course of the dyadic interaction. Then, we calculate the entropy rate for consecutive non-overlapping time windows, small enough to ensure stationarity but not too small, as to ensure convergence. Time windows must also ensure tight 95% confidence intervals in determining Gamma parameters according to MLE. We calculate the entropy rate both for the normalized MMS and for the corresponding binary MMS trains that we obtain by setting peak values to "1" and zero values to "zero".

To estimate the entropy rate we used Approximate entropy ApEn (developed by Steve M. Pincus [57]), which measures the amount of regularity or unpredictability of fluctuations over time-series data that have lengths compatible with experimental settings (unlike other measures of entropy aimed at measuring regularity but requiring very long times). There are caveats to the use of the ApEn algorithm [49]:

i. The ApEn algorithms allows self-counting when counting the number of templates that are similar to a given data segment, which helps avoid the occurrence of $\log(0)$ in the calculation.
ii. However, when the self-similarity threshold r is very small, the template vector coincides only with itself, giving ApEn low values, indicating regularity when the system may in fact, be very irregular.
iii. ApEn is biased by a factor of $\frac{1}{N-m}$, which means that it depends on the template length and data stream length.

ApEn generally depends on the threshold r, and the embedding delay and embedding dimension of the reconstructed space (which is equal to the template length). It is generally suggested, that in order to compare the approximate entropies of different time series, all parameters must be equal. However, for the scope of our study, we chose the threshold parameter r to be equal to 0.2σ, as recommended in the literature [49]. The embedding delay was chosen according to the minimum Average Mutual Information criterion, to ensure maximum novelty between consecutive samples in the reconstructed space. As for the template, we chose it to be $1/R$, where R is the average rate (frequency) of MMS in the time window of interest. This equals to the average time-distance between two spikes and our choice ensures that in the reconstructed space, the coordinates of a point in time include both zeros ("*quiet moments*") and activity spikes and that the system does not bounce back and forth from a single coordinate of zeros components. In this way, we can minimize any bias introduced by differences in

spike rates, in the computation of self-similarity by the algorithm. Sparser time windows will contain the same percentage of "*active*" moments as denser time windows. Since it turns out that, for our datasets, $0.1 < R < 0.5$, we have $2 < m < 10$, which according to the literature is within the optimal range [49]. Moreover, since $N = 1000$, the bias introduced by m in the prefactor is very small.

ApEn is computationally efficient. One can easily see that the worst-case time complexity of ApEn is $O(N^2)$. Furthermore, it has lower effect from noise in the data. If data is noisy, the ApEn measure can be compared to the noise level in the data to determine what quality of true information may be present in the data [49]. We here notice the difference between the criterion for randomness in the Gamma parameter space, when the shape is 1, which is the special case of the memoryless exponential distribution. In our empirical characterization of the MMS from the peak fluctuations, which follow a scaling power law, as the shape approaches the value of 1 representing the exponential distribution case, the $NSR = log\theta$ approaches its maximum levels [3]. The differential entropy for the Gamma distribution has the general form in Equation 2.33, [58]:

$$h(X_g) = k + log\theta + \log\Gamma(k) + (1-k)\psi(k) \qquad \text{Equation (2.33)}$$

We will show later that discrete samples X_G that follow the Gamma distribution, such as the MMS, have entropy roughly equal to $h(X_g) - log\Delta$, when Δ, is the discretization step. Because of the Power Law discussed before, $log(k) = a + b\, log(\theta) + \varepsilon$, we have in Equation 2.34:

$$H(X_G) \cong e^{a+\varepsilon}e^{bNSR} + NSR + \log\Gamma(k) + (1-k)\psi(k) - log\Delta \qquad \text{Equation (2.34)}$$

As the NSR increases, $k \to 1$ and thus, $H(X_G) \to e^{a+\varepsilon}e^{bNSR} + NSR + 1 - log\Delta$. In Section 4, we will experimentally show that, for $log\theta < 1$ in Equation 2.35:

$$H(X_G) = O(NSR), k \to 1 \qquad \text{Equation (2.35)}$$

However, in the case of ApEn, we consider a process of the form $X_G * X_B^t$, where X_B^t is the binary spike series, determining the temporal distribution of the peaks in time. In fact, we will empirically demonstrate that X_B^t is almost independent from X_G, implying that ApEn measures the information content of the binary spike time series, characterizing the motor code. On the other hand, randomness in the sense of NSR (or equivalently $H(X_G)$), refers to the temporal component of the events and answers the question of predictability (future events) in time, whereby predicting future events in time does not benefit from knowledge of prior or current event times. We will see later that these two elements of the Gamma distributed MMS are indeed separable and within the current context, tend to be orthogonal.

In this sense, we propose that the entropy rate (ER) derived from ApEn is a measure to characterize *motor autonomy* in the system. Since ER is a proper way to quantify regularity *vs.* randomness, we can safely presume that the information levels that it carries also measures the ability of an observer to predict the motor behavior of an agent, when the two of them engage in a dyadic social interaction. For example, when the clinician observes the behavior of a child that engages in repetitive and predictable motions, they can easily learn to predict their behavioral and motor patterns. This also implies that they can more easily detect when the child behaves predictably in a certain way and set up the context to better control the situation. Perhaps the child is in distress trying to self-sooth through repetitive movements. In this sense, the more predictable the situation is, the more control it will be afforded by the external agent to *e.g.*, help regulate the child.

2 Clinical Aspects of the Study

2.1 The Autism Diagnostic Observation Schedule (ADOS-2) Scoring System

The ADOS-2 Modules consist of tasks that the clinician performs with the child to observe behavior related to the diagnosis of ASD and reach a conclusion. There are different Modules. Each child is administered a single Module based on their expressive language level, developmental age and their unique interests and abilities. However, they are designed in such a way that ensures that judgements about social and communicative abilities are as independent as possible from level of language ability and chronological age.

Both Modules (toddler) T and 1 are administrated to non-speaking children, Module T for ages 12-30 months and Module 1 for children over 31 months. Module 2 is administrated to children of all ages who are using phrase speech but are not yet speaking with fluency. Modules 3 and 4 are administrated to individuals that are speaking with verbal fluency, with Module 3 specifically designed for Children / Younger adolescents that can still play with action figure-type toys and Module 4 for older adults. All Modules are administrated under the assumption that the individual can walk independently, has no motor issues and is free of visual or hearing impairments. These assumptions are erroneous, as they do not consider known prolonged temporal delays in sound processing since birth [18] or subtle involuntary motor control issues present across the spectrum of autism and ADHD [13; 36; 38; 59; 60]. However, we use the ADOS-2 test not to diagnose but to evoke social situations leading to movement patterns likely present in such situations.

Our current analysis focuses on Modules 1,3 and 4. We suggest the following categorization of tasks to better relate our digital biomarkers to the clinical tasks that evoke some aspect of social interactions and emotions present in human gestural communication, which is mediated by movements:

Socio-Motor Tasks: These are tasks that engage interactive movements within the child, the clinician, and jointly between the child and clinician. Construction Task, Joint Interactive Play, Demonstration Task, Cartoons, Conversation and Reporting and Break Tasks all have in common the Child's Socio-

Motor behavior involvement. Construction Task consists of an interaction between the Clinician and Child that involves reaching over the Clinician's arm to ask for block pieces that may form a shape. Joint Interactive Play consists of a Play Sequence between the Child and the Clinician that involves body movements. During Demonstration Task the Child uses their body to represent objects and mime the use of each object. During Cartoon Task, the Clinician observes the Child's gestures and coordination with speech. Similarly, during Conversation and Reporting body language and facial expressions / gestures are observed alongside general communicative skills. During Break the Child is expected to move around the room, perhaps eat a snack or drink water, etc.

Emotional Tasks: These are tasks that probe the child's emotional states. Emotions, Social Difficulties, Friends, Relationships, and Marriage and Loneliness all evoke strong emotional responses from the Child. During the Emotions Task, the Child is asked questions about social relationships, different emotions such as happiness, fear and anxiety and details about the manifestation of these emotions under different circumstances. Social Difficulties and Annoyance consist of questions related to social interactions at school or work, such as bullying or teasing. Friends, Relationships, and Marriage are designed to evaluate the Child's concepts on topics such as friendship and social relationships and the questions asked can cause strong emotions in the Child. Similarly, during Loneliness task, questions are asked about the concept of loneliness, which is a heavy topic, especially for Children on the Autism Spectrum, that struggle with social rejection and bullying from a young age.

Abstract Tasks: These are tasks that require higher, abstract level of cognition. Make-Believe Play, Description of a Picture, Telling Story from a Book, and Creating a Story all help observe higher cognitive skills. Make-Believe Play involves the use of dolls an action figures and the Child is tested for their ability to perceive them as animate beings and produce imaginative sequences of actions that involve these objects. Perception or the lack of it of objects as animate beings is a concept frequently encountered within the context of the Theory of Mind. During Description of a Picture Task the Clinician observes the Child's use of language/ communication and the level of interest in the picture presented. Telling a Story from a Book is similar but involves a story from a book instead of a picture and humor and presumption of the feelings of the characters from the book are evaluated as well.

After the administration of Module 3 a scoring system is used to evaluate the levels of Social Affect (SA) and Restricted and Repetitive Behavior (RRB). The scores are added up to determine a final score, from 0 to 10. A score of 0 or 1 indicates Minimal to No Evidence of ASD related symptoms, scores between 2 and 4 indicate a Low Level of symptoms related to ASD, 5 to 7 Moderate and 8 to 10 High. A score of 9 or more determine that the Child is Autistic whereas a score of 7 of more that the Child is in the Autism Spectrum. Furthermore, Social Affect consist of Communication (Reporting of Events, Conversation and Descriptive, Conventional, Instrumental, or Informational Gestures) and Reciprocal Social Interaction (Unusual Eye Contact, Facial Expression Directed to Examiner, Shared Enjoyment in Interaction, Quality of Social Overtures, Amount of Reciprocal Social Communication, Overall Quality of Rapport) Scorings. RRB consists of scoring Stereotyped/ Idiosyncratic Use of Words or Phrases, Unusual Sensory Interest in Play Material/ Person, Hand and Finger and Other Complex Mannerisms and Excessive Interest or Highly Specific Topics/ Objects or Repetitive Behaviors.

3 Supplementary Figures

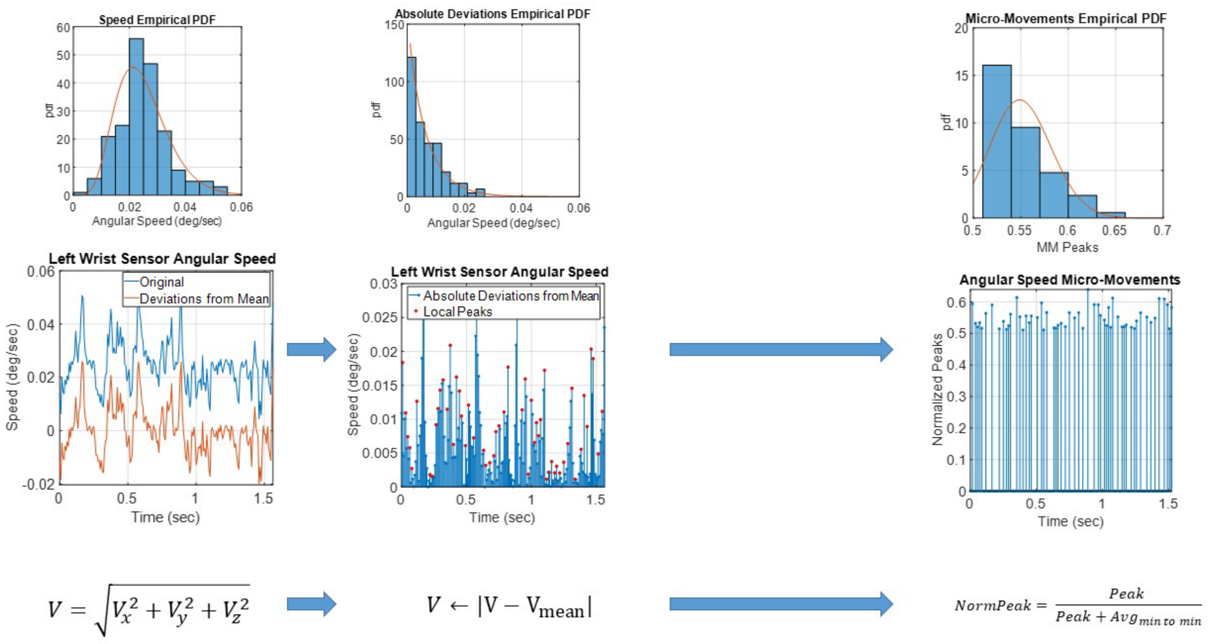

Supplementary Figure 1. The Micro-Movement Spikes (MMS) computation pipeline. First, the empirical mean is obtained from the peaks in the original time series data, using maximum likelihood estimation (MLE). Then, absolute deviations from the empirical mean are obtained and the time series of these deviations used. Here notice that the time stamps of the original time series are retained, as all points in the original time series are used. The local maxima (peaks) are then normalized by their local average. This is obtained from the average of all points between the two adjacent local minima (as in the formula over the arrow). Finally, we obtain the standardized MMS series. This is a scalar value at each point of the time series which has now been corrected for allometric effects due to anatomical disparities across participants. The normalized quantity allows us to compare the fluctuations in motion parameters across children of different ages and different body sizes. Importantly, the 0-values in the MMS are “quiet moments” of the body part being measured, representing baseline average activity (according to the empirically estimated mean value), while the values of the MMS away from 0 represent bursts of activity above the baseline average activity of the person and context. In this sense, the MMS allow for a personalized approach to movement analysis and provide the means to obtain nuance information for the same type of motion (for the same motor program being learned or being deployed) across different contexts and situations.

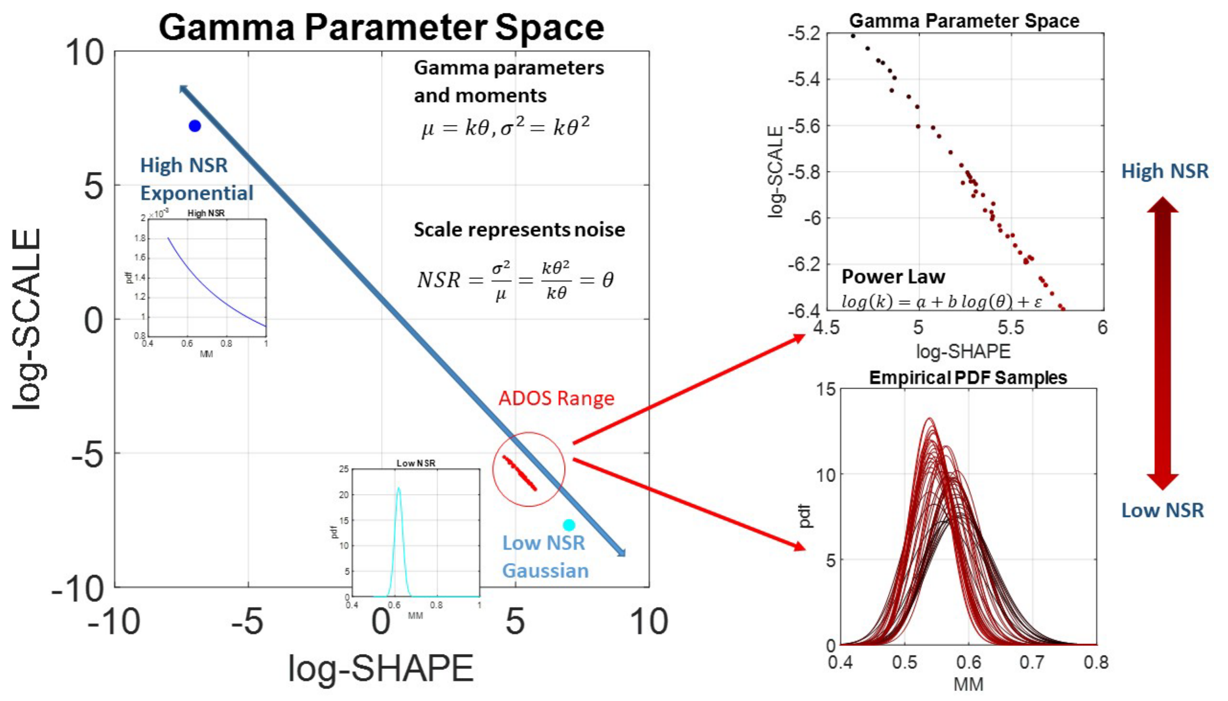

Supplementary Figure 2. The standardized Micro-Movement Spikes are best fit by the continuous Gamma family of probability distributions found to bests characterize the moment-by-moment fluctuations in human motion (in an MLE sense). The shape and the scale parameters of the Gamma distribution from the MMS follow a power law relationship, whereby knowing the scale predicts the shape, and vice versa. This scaling relation on the log-log Gamma parameter plane, has been confirmed by different studies assessing a multitude of biorhythmic parameters, spanning from the autonomic, reflexive, involuntary, spontaneous, automatic and voluntary levels of motor function. In the Gamma family, the scale parameter is equal to the Noise-to-Signal ratio, the NSR (the Gamma variance over the Gamma mean). For stochastic regimes of low NSR, the distribution asymptotically converges to a Gaussian like symmetric shape, whereas for systems with high noise, the distribution tends to the memoryless Exponential regime. While in the former prior information can be predictive of present or even future events, in the latter, the information is in "the here and now" with no predictive power of prior events towards present or future events.

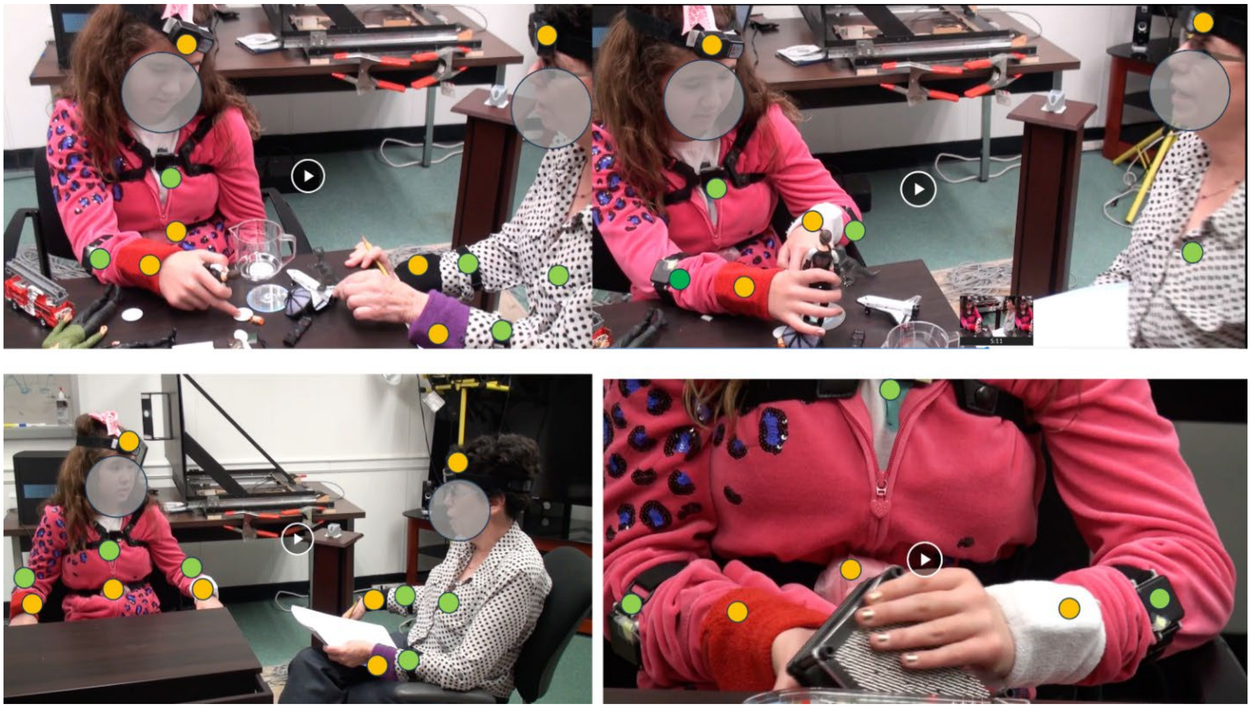

Supplementary Figure 3. Sensors positioning on the child and clinician. Sensors represented in green are the APDM Opals, Portland Oregon, sampling at 128Hz. They output triaxial acceleration through inertial measurement units (IMUs), triaxial angular velocity (through a gyroscope) and temperature through temperature sensors. The data from the gyroscopes was used in the present study, using the angular speed time series. The orange sensors are from the Polhemus Liberty Latus (Colchester, Vermont) sampling at 188Hz. We did not use these data in the present study. The four scenes are frames from the video recording during the ADOS performance. They reflect different tasks and conversational segments of the research grade ADOS test.
