# Supplementary figures and images for: Digital screener of socio-motor agency balancing motor autonomy and motor control
Source: Front Hum Neurosci. 2024 Oct 1;18:1442799. doi: 10.3389/fnhum.2024.1442799 (PMC11473353; doi:10.3389/fnhum.2024.1442799)

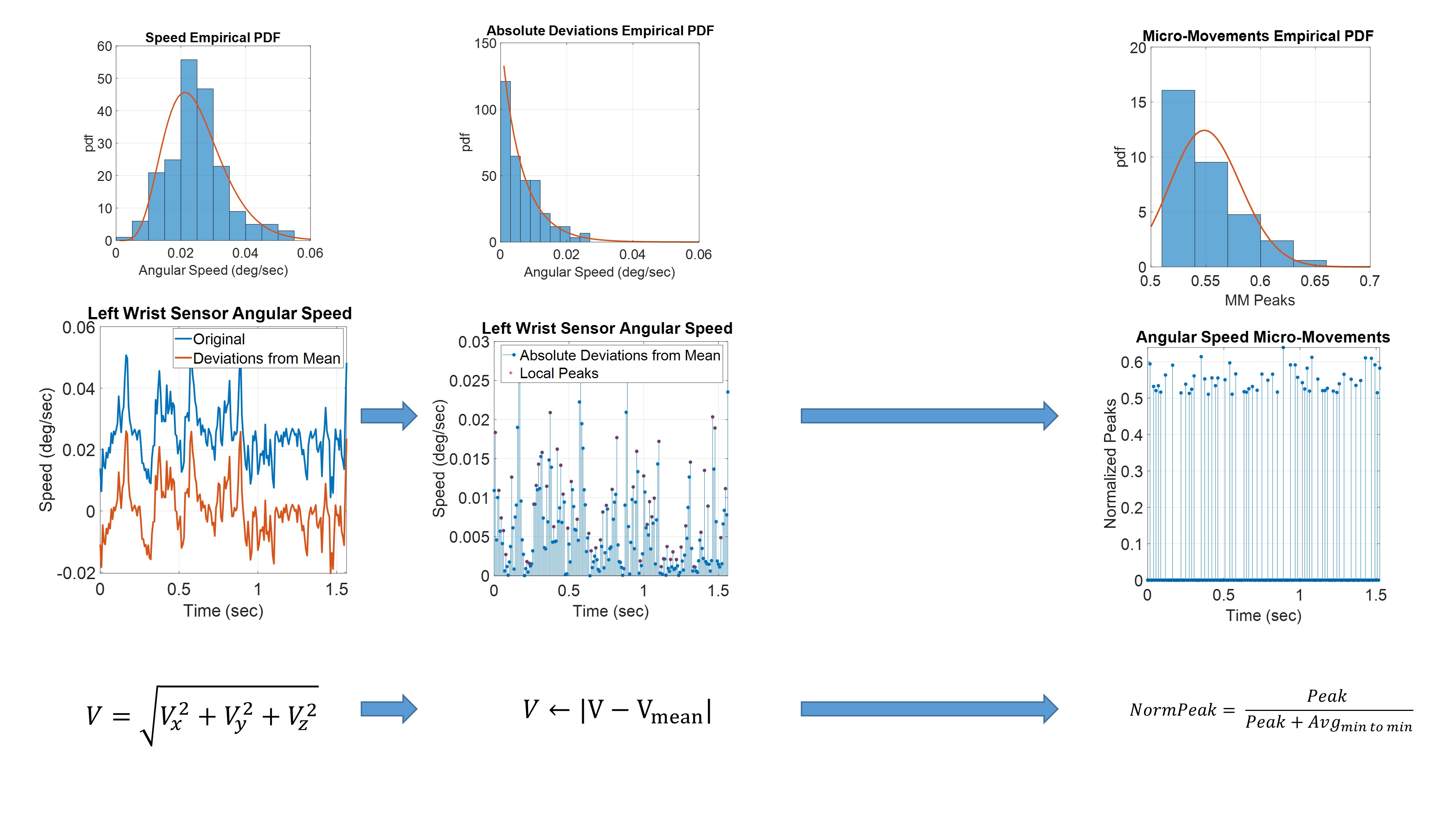

Supplement: Supplementary file 2 [file Presentation_1.zip › SM_1442799/Supplementary_Figure1.jpg]

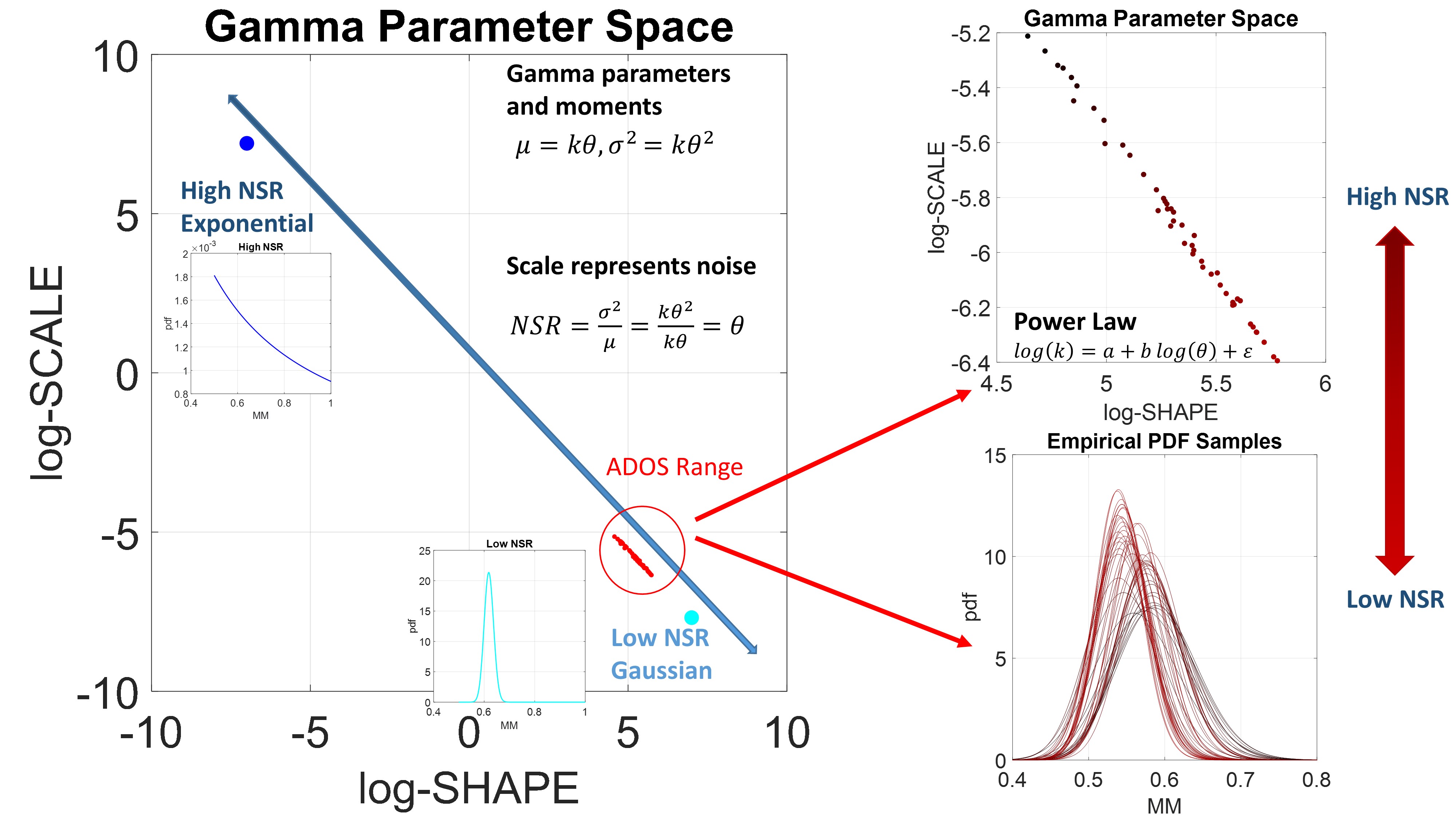

Supplement: Supplementary file 2 [file Presentation_1.zip › SM_1442799/Supplementary_Figure2.jpg]

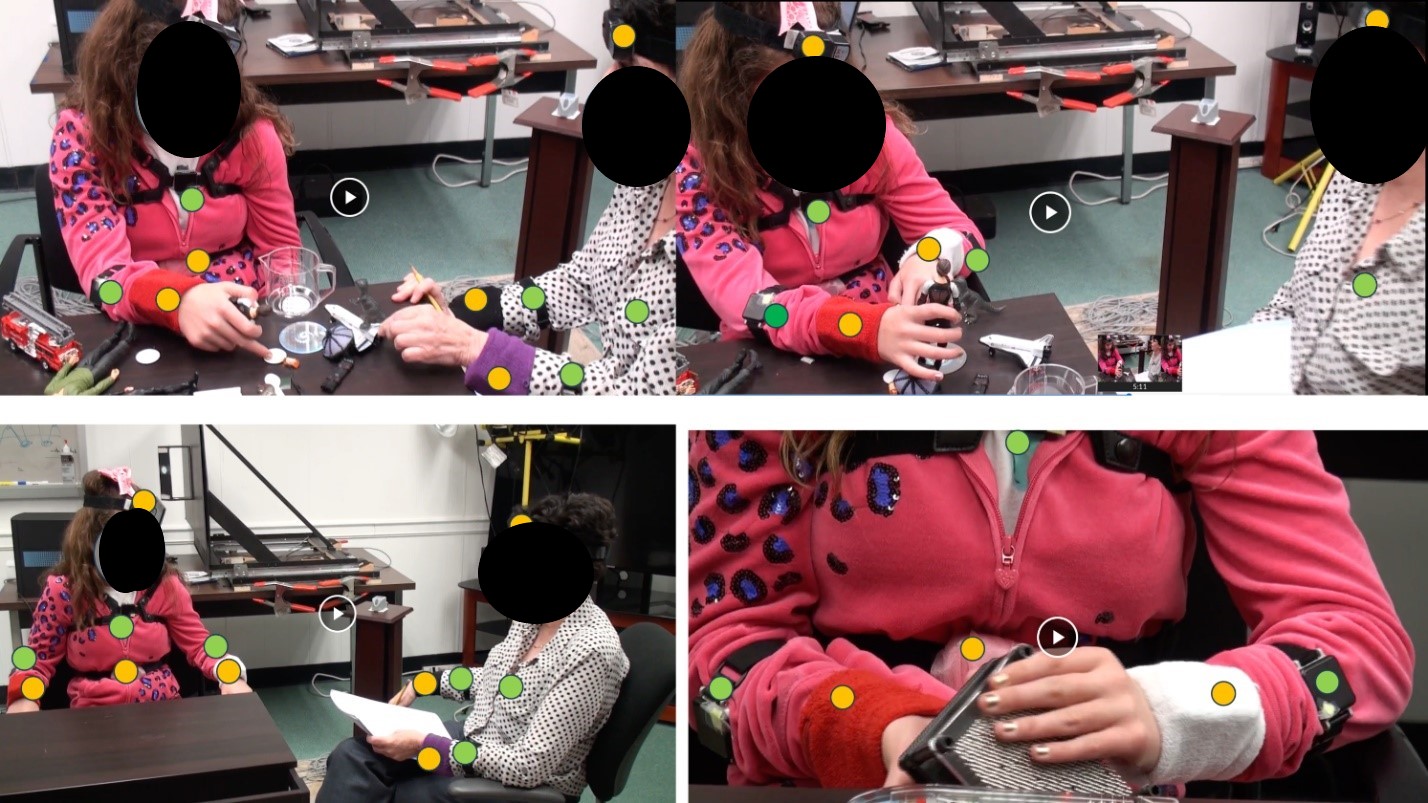

Supplement: Supplementary file 2 [file Presentation_1.zip › SM_1442799/Supplementary_Figure3.jpg]
